# Bioorthogonal non-canonical amino acid tagging reveals translationally active subpopulations of the cystic fibrosis lung microbiota

**DOI:** 10.1038/s41467-020-16163-2

**Published:** 2020-05-08

**Authors:** Talia D. Valentini, Sarah K. Lucas, Kelsey A. Binder, Lydia C. Cameron, Jason A. Motl, Jordan M. Dunitz, Ryan C. Hunter

**Affiliations:** 1 Department of Microbiology & Immunology, University of Minnesota, 689 23rd Avenue SE, Minneapolis, MN 55455 United States; 2 Academic Health Center, University Flow Cytometry Resource, University of Minnesota, 6th St SE, Minneapolis, MN 55455 United States; 3 Division of Pulmonary, Allergy, Critical Care & Sleep Medicine, University of Minnesota, 420 Delaware St. SE, Minneapolis, MN 55455 United States

**Keywords:** Microbial ecology, Microbiome

## Abstract

Culture-independent studies of cystic fibrosis lung microbiota have provided few mechanistic insights into the polymicrobial basis of disease. Deciphering the specific contributions of individual taxa to CF pathogenesis requires comprehensive understanding of their ecophysiology at the site of infection. We hypothesize that only a subset of CF microbiota are translationally active and that these activities vary between subjects. Here, we apply bioorthogonal non-canonical amino acid tagging (BONCAT) to visualize and quantify bacterial translational activity in expectorated sputum. We report that the percentage of BONCAT-labeled (i.e. active) bacterial cells varies substantially between subjects (6-56%). We use fluorescence-activated cell sorting (FACS) and genomic sequencing to assign taxonomy to BONCAT-labeled cells. While many abundant taxa are indeed active, most bacterial species detected by conventional molecular profiling show a mixed population of both BONCAT-labeled and unlabeled cells, suggesting heterogeneous growth rates in sputum. Differentiating translationally active subpopulations adds to our evolving understanding of CF lung disease and may help guide antibiotic therapies targeting bacteria most likely to be susceptible.

## Abstract (web summary)

Bioorthogonal non-canonical amino acid tagging (BONCAT) coupled with flow cytometry (FACS) has been utilized to profile active microbiome of environmental samples. Here, the authors combine BONCAT-FACS and 16 S rRNA sequencing to characterize the translational activity of bacterial communities within sputum derived from stable cystic fibrosis patients.

## Introduction

The increased viscosity and impaired clearance of mucus secretions in cystic fibrosis (CF) airways creates a favorable environment for chronic microbial colonization, the primary cause of morbidity and mortality^1^. *Pseudomonas aeruginosa* and *Staphylococcus aureus* have long been recognized as primary CF pathogens and are the targets of common therapeutic regimens^2^, though recent culture-independent studies have revealed a more complex polymicrobial community harboring facultative and obligately anaerobic bacteria that are relatively understudied^3–5^. While the specific contributions of individual community members to disease progression remain poorly understood and at times controversial^6^, cross-sectional studies of both pediatric and adult cohorts have revealed compelling relationships between bacterial community composition and disease stage, antibiotic use, age, and other phenotypes^7–12^. These data have challenged the field to reconsider therapeutic strategies in a polymicrobial community context^13,14^.

Relatively fewer studies have identified within-subject perturbations in bacterial community structures that coincide with acute and complex disease flares known as pulmonary exacerbations (PEx). Though no standardized definition of PEx is broadly accepted^15^, these episodes are generally characterized by increased respiratory symptoms (e.g., shortness of breath, sputum production) and acute decreases in lung function that can, but not always, be resolved in response to antibiotic therapy. While this would suggest a bacterial etiology, sputum cultures generally demonstrate that airway pathogens are recovered at similar densities before, during, and after disease flares^16–19^. Culture-independent studies show similar trends; with exceptions^9,20–22^, longitudinal sequencing analyses of sputum from individual subjects frequently reveal unique, subject-specific bacterial communities whose diversity and composition remain stable during PEx onset and upon resolution of disease symptoms^16,23,24^. This lack of association between lung microbiota and disease dynamics may reflect the inability of both culture-based and sequencing approaches to capture changes in bacterial activity, which likely have a critical impact on disease progression and therapeutic effectiveness.

To date, there have been few studies of bacterial growth and metabolism within the CF airways^25–30^. RNA-based profiling of stable CF subjects has shown consistencies between RNA and DNA signatures suggesting that many bacterial taxa identified by 16 S rRNA gene sequencing are metabolically active, though these data have also corroborated that bacterial community membership is not necessarily predictive of growth activity^25,26^. Further, rRNA/DNA ratio methods are inherently constrained for use on complex bacterial communities with varying growth strategies (i.e., human microbiota)^31,32^. Interactions between respiratory pathogens and the host and/or co-colonizing microbiota can influence growth rates, metabolism, virulence factor production, and antimicrobial susceptibility without an accompanying change in bacterial abundance^33–38^. And finally, growth rates of respiratory pathogens can vary substantially between subjects and even within a single sputum sample^27,28^, the heterogeneity of which is not captured using conventional molecular profiling. There remains a need for novel methods to characterize bacterial activity and its role in disease progression.

Bioorthogonal non-canonical amino-acid tagging (BONCAT) has been used to characterize the activity of uncultured microbes in soil and marine samples^39–43^. BONCAT relies on the cellular uptake of a non-canonical amino-acid (e.g., L-azidohomoalanine (AHA), a L-methionine analog) carrying a chemically-modifiable azide group^44^. After uptake, AHA exploits the substrate promiscuity of methionyl-tRNA synthetase and is incorporated into newly synthesized proteins. Translationally active cells can then be identified through a bioorthogonal azide-alkyne click reaction in which a fluorophore-tagged alkyne is covalently ligated to AHA, resulting in a fluorescently labeled population of translationally active cells that can be further studied using a variety of microscopy and analytical methods. BONCAT has been shown to correlate with other established methods of quantifying microbial activity^40,43^ and represents a robust tool for characterization of bacterial communities and a range of other organisms in their native growth environment.

BONCAT has also recently been used to study bacterial pathogens in vitro^45–48^, though it has seen limited use in the study of host-associated bacterial communities^40,49^. Samples derived from the CF airways provide a unique opportunity to do so, as the site of infection is amenable to longitudinal studies and the bacterial growth environment is relatively stable upon removal from the host^50^. Exploiting these advantages, here we use BONCAT together with imaging, fluorescence-activated cell sorting (FACS) and 16 S rRNA gene sequencing to characterize the translational activity of bacterial communities within sputum derived from a cohort of seven clinically stable CF subjects. We reveal that active bacteria represent only a subset of microbiota captured using conventional 16 S rRNA gene sequencing and discuss these results in the context of progression and treatment of chronic airway disease.

## Results

### BONCAT differentiates translationally active bacteria

**Fig. 1:**
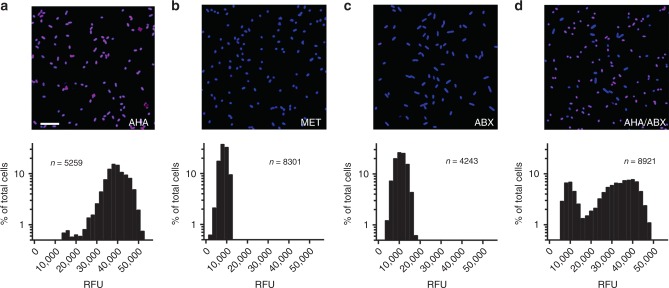
BONCAT labeling of *P. aeruginosa* differentiates translationally active and inactive cells. *P. aeruginosa* was incubated in the presence of **a** AHA, **b** methionine (MET), and **c** antibiotics prior to AHA (ABX). Actively growing cells were identified via strain-promoted click chemistry (Cy5, magenta; SYTO64, blue). Histograms associated with each image represent average Cy5 pixel intensity (relative fluorescence units, RFU) per cell. **d** Two AHA-treated cultures (one with antibiotics, one without) were mixed in a 1:1 ratio prior to Cy5–DBCO labeling. These data demonstrate that BONCAT can differentiate translationally active and inactive bacterial cells in a complex nutritional milieu. Scale bar = 10 µm. *n* refers to the number of cells examined over ten images from each of three independent experiments. Source data are provided as a Source Data file.

To optimize the BONCAT experimental approach, we first grew *P. aeruginosa*, a canonical CF pathogen, to mid-log phase followed by supplementation with 6 mM L-azidohomoalanine (AHA) for 3 h. Post-AHA treatment, azide-alkyne click chemistry using Cy5-labeled dibenzocyclooctyne (Cy5–DBCO), permitted fluorescent detection of translationally active cells (Fig. 1a). Quantification of average Cy5 pixel intensity per cell revealed active protein synthesis in ~98% of the population. By contrast, supplementation of the growth medium with 6 mM L-methionine (MET) or pretreatment of *P. aeruginosa* with tobramycin, chloramphenicol, and tetracycline (to arrest de novo protein synthesis) prior to AHA resulted in negligible fluorescence (Fig. 1b, c). These data were also confirmed by SDS-PAGE (Supplementary Fig. 1). Finally, when two AHA-labeled cultures (one treated with antibiotics, one without) were combined in a 1:1 ratio prior to Cy5–DBCO labeling, a bimodal distribution of fluorescence intensities were observed, representing a mix of active and inactive cells (Fig. 1d). Together, these data demonstrate the utility of BONCAT for characterizing *P. aeruginosa* translational activity in an amino-acid-rich growth environment.

**Fig. 2:**
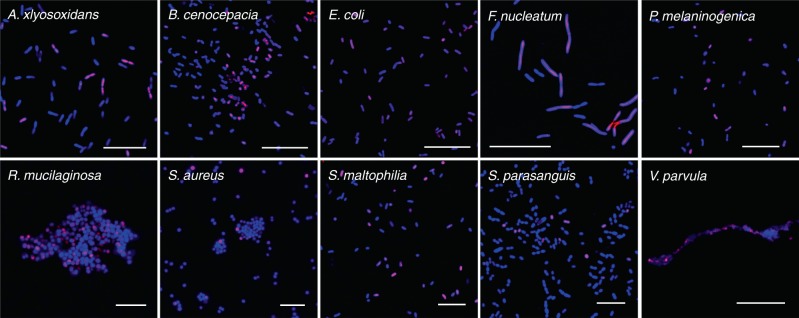
BONCAT can identify active cells among diverse CF microbiota. Two cultures (one treated with antibiotics, one without) of each species were grown in the presence of AHA and mixed 1:1 prior to Cy5–DBCO (magenta) labeling and SYTO64 counterstaining (blue). These data demonstrate that BONCAT can differentiate between active and inactive bacterial cells among diverse CF microbiota. Scale bars; *Ax, Bc, Fn, Ec, Pm, Rm* = 20 µm; *Sa, Sm, Sp* = 10 µm; *Vp* = 5 µm. Images are representative of ten images from each of three biologically independent experiments for each organism.

To assess whether BONCAT is broadly suitable for labeling polymicrobial communities found in the airways, we then performed mixed activity labeling as described above on representative isolates of common CF-associated microbiota;^51^ *Achromobacter xylosoxidans, Burkholderia cenocepacia, Escherichia coli, Fusobacterium nucleatum, Prevotella melaninogenica*, *Rothia mucilaginosa*, *Staphylococcus aureus*, *Stenotrophomonas maltophilia*, *Streptococcus parasanguinis*, and *Veillonella parvula* (Fig. 2). Each mixed culture (+/− antibiotics in a 1:1 ratio) exhibited a similar labeling pattern to *P. aeruginosa*, suggesting that BONCAT can be used to characterize translational activity among diverse bacterial taxa associated with the CF airways. Notably, all species tested demonstrated BONCAT labeling. In addition, AHA did not affect the growth phenotype of any species under our experimental conditions (Supplementary Fig. 2), consistent with previous studies showing that BONCAT permits labeling of microbiota without concomitant changes in growth rate or protein expression^40,52^.

### BONCAT identification of active CF microbiota

The BONCAT protocol optimized for lab-grown cultures was then modified for analysis of CF bacterial communities in sputum. To do so, sputum was collected from clinically stable subjects and immediately supplemented with cycloheximide to reduce AHA incorporation by host cells (Supplementary Fig. 3). Samples were then divided into three equal-volume aliquots, supplemented with either 6 mM AHA, 6 mM methionine, or antibiotics (30 µg mL^−1^ chloramphenicol, 200 µg mL^−1^ tetracycline, and 10 µg mL^−1^ tobramycin) plus 6 mM AHA, and incubated at 37 °C under oxic conditions for 3 h. Incubation time was chosen to maximize labeling while minimizing changes in bacterial growth conditions such that they closely reflected the in vivo chemical environment. AHA concentration (6 mM) was based on average methionine content in CF sputum (0.6 mM)^53^ and a 10:1 AHA:MET ratio (or greater) required for effective labeling (Supplementary Fig. 4).

**Fig. 3:**
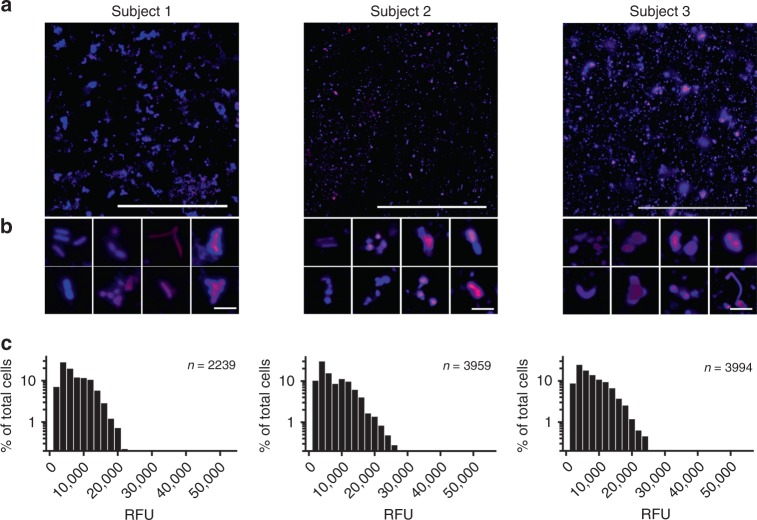
CF microbiota exhibit heterogeneous translational activity within sputum. **a** Sputum was incubated in the presence of 6 mM AHA immediately upon expectoration. BONCAT labeling with Cy5–DBCO (magenta) and counterstaining with SYTO64 (blue) reveals heterogeneous AHA incorporation (i.e., translational activity). **b** Higher magnification images further emphasize the range of bacterial activity at the single-cell level. **c** Average Cy5 pixel intensity per cell suggests slow and heterogeneous translational activity among bacterial cells in situ. Scale bars; **a** = 100 µm, **b** = 5 µm. Source data are provided as a Source Data file.

Representative micrographs (Fig. 3a) reveal BONCAT-labeled sputum obtained from three individual CF subjects (Supplementary Table 1, subjects 1–3). Consistent with previous reports of heterogeneous growth rates^27,28,54^, notable differences in Cy5 fluorescence are apparent at higher magnification (Fig. 3b); several individual cells and cell aggregates show moderate to intense labeling whereas others are unlabeled. Treatment with methionine instead of AHA did not result in fluorescent signal, ruling out non-specific labeling and residual dye that could not be removed by washing (Supplementary Fig. 5). Similarly, treatment of sputum with antibiotics prior to AHA addition also resulted in a significant reduction in fluorescence intensity. However, this reduction was incomplete, which may reflect the development of antimicrobial tolerance that arises among CF pathogens. Finally, though cycloheximide treatment results in a significant reduction in AHA uptake by macrophages (Supplementary Fig. 3), we note that host-cell contributions to BONCAT fluorescence cannot be ruled out. Despite this possibility, average pixel intensity per cell (Fig. 3c) further emphasizes the range of bacterial translational activity and the likely slower growth rates of CF microbiota in sputum compared to cultures grown in vitro (compare Fig. 3c and Fig. 1a). These analyses demonstrate that BONCAT labeling can be used to characterize bacterial activity within complex sputum samples. Moreover, these data suggest that translationally active bacteria represent only a subpopulation of the CF lung microbiota.

### Flow cytometric analysis of BONCAT-labeled CF microbiota

**Fig. 4:**
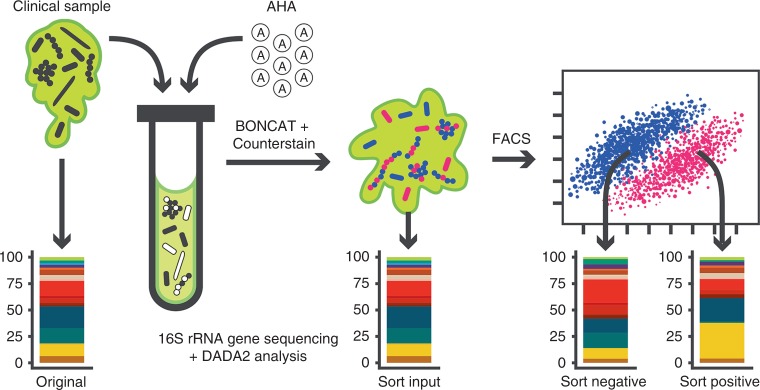
Experimental workflow for BONCAT. Analysis of CF sputum.

BONCAT combined with fluorescence-activated cell sorting (FACS) has previously been used to study microbial activity within soils and marine sediments^39,42^. We therefore sought to use FACS to characterize and isolate BONCAT-labeled (i.e., active) cells and bacterial aggregates within sputum samples derived from clinically stable CF subjects (Supplementary Table 1, Subjects 4–6). Our experimental workflow is shown in Fig. 4. Upon sputum collection, a small aliquot (original) was removed and stored at −80 °C prior to conventional 16 S rRNA gene amplicon analysis. Remaining sputum was then treated with cycloheximide and divided into four aliquots, three of which were supplemented with AHA (6 mM). As a control, the remaining aliquot was treated with 6 mM methionine, and all samples were then incubated under oxic conditions at 37 °C for 3 h. Samples were subjected to Cy5–DBCO labeling and counterstaining, followed by removal of another aliquot (sort input) to determine community profile changes as a result of chemical fixation and sputum incubation ex vivo. Remaining samples were homogenized and filtered to remove host cells, followed by FACS to isolate Cy5− (sort-negative) and Cy5+ (sort-positive) cells.

**Fig. 5:**
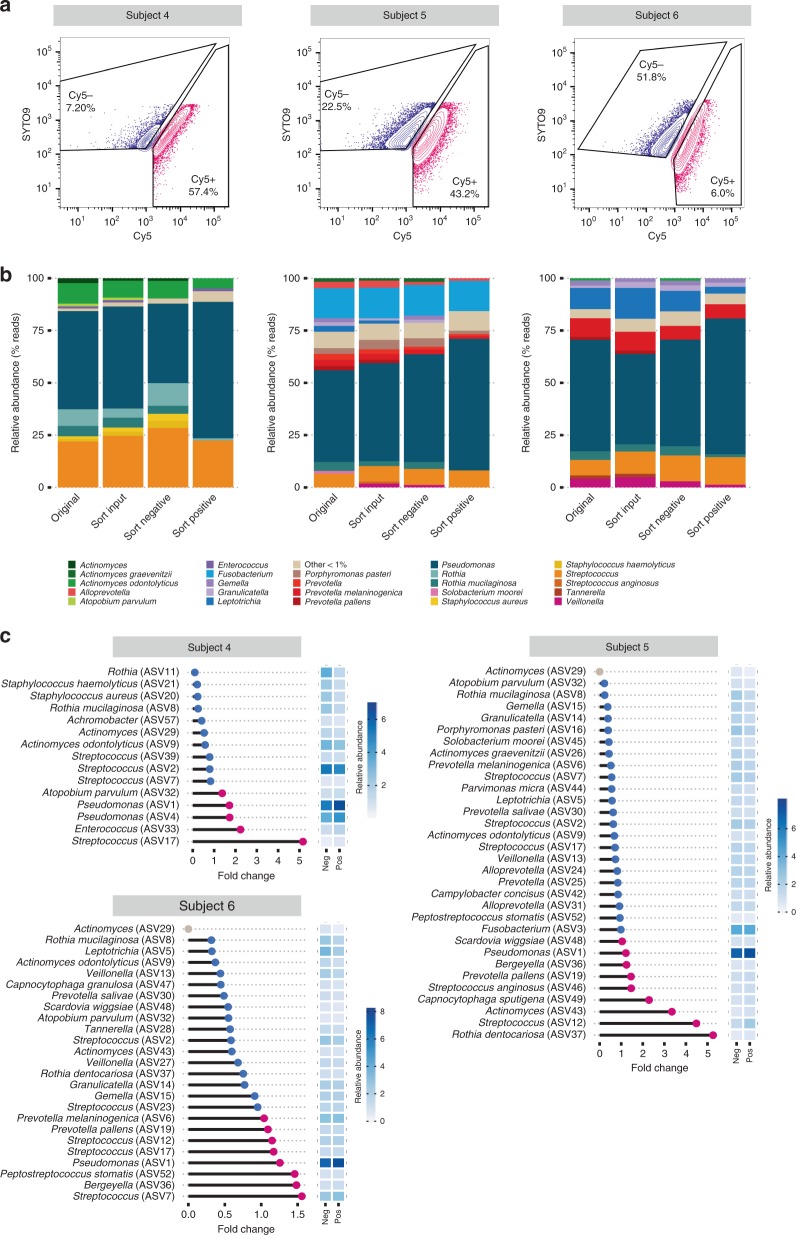
BONCAT, FACS, and sequencing of CF sputum reveals the taxonomic identities of translationally active microbiota. **a** FACS of BONCAT-labeled sputum reveals Cy5− and Cy5+ subpopulations. Percentages shown reflect % of parent population post-CD45RO gating. **b** Original, sort input, sort-negative (Cy5−), and sort-positive (Cy5+) fractions were analyzed by 16 S rRNA gene sequencing. Taxa plots summarize sequencing data by subject and averaged relative abundances between triplicate-positive and -negative sorted fractions. **c** Fold-changes between relative abundances of taxa in the sort-positive compared to the negative fraction. Point color indicates taxa that were increased (pink) and decreased (blue) in relative abundance in the sort-positive fraction, representing translationally active microbiota. The single gray points indicate ASVs seen only in the negative sample. Heatmap sidebars represent square root transformed relative abundances.

Given the potential heterogeneity of a single sputum plug, triplicate aliquots from each CF sputum sample were BONCAT-labeled and analyzed by FACS and 16 S rRNA gene sequencing to assess the consistency of results (Supplementary Figs. 7 and 8). Representative FACS plots are shown in Fig. 5a. Cy5− and Cy5+ gates were sample-specific and were established first by using an AHA− (MET+) aliquot to define the negative gate for each sample. Positive gates were then conservatively assigned by comparing the AHA+ aliquots to the AHA− control (see Supplementary Fig. 9 for gating scheme). AHA+ samples underwent notable shifts along the Cy5+ axis (Fig. 5a) and cells that fell within the Cy5+ gate exhibited a higher geometric mean of fluorescence intensity in the Cy5 channel (Supplementary Fig. 10). These data confirm BONCAT labeling and are reflective of translational activity (Fig. 5a). Sort specificity was validated by immunostaining using an anti-Cy5 antibody, which revealed estimated false-negative and false-positive rates of 6.8% and 12%, respectively (Supplementary Fig. 6). Based on total events (~6.5 million counts per sample, on average; Supplementary Table 2), we consistently found that only a subset of the overall bacterial population was Cy5+. For the three individuals surveyed, replicate averages of the Cy5-labeled population were 6.2% (+/− 1.21), 42.6% (+/− 5.62), and 56.1 (+/− 9.57) for subjects 4, 5, and 6, respectively. These data reflect labeling patterns shown by microscopy (Fig. 3) and suggest that expectorated sputum harbors bacterial communities with a range of translational activity.

### Taxonomic identities of active sputum microbiota

16 S rRNA gene sequencing was applied to original, sort input, sort-negative, and sort-positive fractions from each sample to determine bacterial community composition. Sequence data were analyzed using the DADA2 pipeline^55^ to reduce potential loss of biological sequence variation due to clustering by similarity and to improve observation of fine-scale variation (including species-level resolution) in bacterial populations. Using this approach, sequence data derived from AHA-labeled samples (sort input, sort-negative, and sort-positive) were compared to their paired original sample to characterize translationally active subpopulations. For each subject, sample replicates were compared by proportions of the top-ranking taxa (Supplementary Fig. 8a), and variation related to sample type was visualized using a double principle co-ordinates analysis (DPCoA) (Supplementary Fig. 8b). Replicate samples showed considerable agreement, thus, relative abundances were averaged for further analysis.

Each subject harbored lung microbiota of low to moderate complexity (Fig. 5b), and community profiles were consistent with prior 16 S rRNA gene surveys of CF sputum in which *Pseudomonas* and *Streptococcus* were dominant genera^3–5,9–12^. We also achieved species-level resolution for less abundant taxa, including several obligate and facultative anaerobes (e.g*., Prevotella sp., Rothia sp*.). In general, AHA labeling did not result in substantial changes in bacterial membership; for the most abundant taxa (>1%), community composition was comparable before (original) and after (sort input) BONCAT labeling, demonstrating that AHA treatment and chemical fixation had minimal effect on relative bacterial abundance. Interestingly, bacterial populations recovered from BONCAT–FACS analysis (sort-negative, sort-positive) also showed similarities among the most abundant community members relative to the original sample (i.e., those detected by conventional 16 S rRNA gene sequencing). Notable exceptions were *Staphylococcus aureus* and *Rothia sp*. for subject 4, which despite efficient labeling in laboratory culture, were of negligible abundance in the positive fraction, suggesting low translational activity. Less abundant taxa (Supplementary Fig. 11), showed greater variation between fractions, but most were also generally detectable in both sort-negative and sort-positive gates. Together, these data suggest that a subset of most taxa detected by conventional 16 S rRNA gene sequencing are translationally active. Moreover, each taxon appears to exhibit heterogeneous growth activity, which may have important implications for the progression and treatment of CF disease.

To better observe changes in the relative abundances of translationally active bacterial taxa, we calculated fold-change differences between sort-negative, sort-positive, and sort input fractions for each subject (Fig. 5c and Supplementary Figs. 12 and 13). Some genera/species present in fold-change plots do not appear in taxa plots (Fig. 5b) because they were less than 1% relative abundance, but we note that activity among these less abundant populations may also be determinants of CF pathogenesis. In general, ranks of relative abundance were not appreciably different between sort-positive and sort-negative fractions (denoted by heatmaps in Fig. 5c). The most abundant organism in all subjects, *Pseudomonas* (ASV1/ASV4), was always in greater relative abundance in the positive sort (Fig. 5c), reflecting its active growth in sputum and underscoring its recognized importance as a CF pathogen. However, this trend of agreement between relative abundance and fold-change did not always hold. For example, *Leptotrichia* (ASV5) in subject 6 was high in rank abundance (see heatmaps), but its fold difference between positive and negative fractions was 0.22 (~4.5-fold greater in the negative sort), indicating lower relative translational activity than its co-colonizing microbiota. This was also observed for *Streptococcus* (ASV2) in subject 4, which was the second most abundant taxon yet showed low translational activity. Conversely, some low abundance organisms were in higher relative abundance in the positive sort. Most notably, *Streptococcus* (ASV12) and *Rothia dentocariosa* (ASV37) in subject 5 had average relative abundances of 1.3% and 2.3%, respectively, in the negative sort, but were 4.5- and 5.25-fold more prominent in the positive fraction. Other low abundance ASVs assigned as *Actinomyces, Enterococcus, Peptostreptococcus*, and *Capnocytophaga sputigena* showed similar trends, which were also confirmed by additional comparisons between sort input/ sort-positive and sort input/sort-negative fractions (Supplemental Figs. 12 and 13).

Taken together, these BONCAT data reveal the extensive heterogeneity of translational activity among CF microbiota. Each individual harbors a unique bacterial community, though community membership and relative abundance are not necessarily predictive of translational activity. Ultimately, profiling of bacterial communities in this manner may help guide therapeutic strategies by identifying subpopulations of translationally active bacteria.

## Discussion

16 S rRNA gene sequencing has become the gold standard for culture-independent characterization of CF airway bacterial communities. Despite the wealth of data that have emerged regarding the complexity of lung microbiota, we have little understanding of bacterial activity at the site of infection and the specific contributions of individual species to pathogenesis. Expanding on recent studies employing BONCAT as a means of characterizing the ecophysiology of microbial communities in their natural growth environment^39–43^, we use this approach in combination with FACS and 16 S rRNA gene sequencing to shed light on bacterial activity in CF sputum. We demonstrate that only a subset of each taxon is detectable by metabolic labeling. Identification and characterization of this subpopulation is not achievable using conventional sequencing approaches and may provide a more precise representation of relevant microbiota within the CF lung.

BONCAT-based studies of translationally active bacteria challenge our thinking on the microbial ecology of the CF airways. Each subject harbored a unique bacterial community consisting primarily of canonical lung pathogens (e.g., *Pseudomonas*). Consistent with previous studies using RNA-based methods^25,26^, BONCAT–FACS-based sequencing data indicate these most abundant taxa are also active in situ, reinforcing a probable role for these genera in CF pathogenesis. However, we also revealed that low abundance community members not commonly associated with CF lung disease comprise many taxa that exhibited increased abundance in the positive sort, indicating that community membership is not always predictive of translational activity. In conventional 16 S rRNA datasets, rare taxa (i.e., <1%) can be challenging to detect among high abundance organisms (or they are commonly grouped into an ‘other’ category). Moreover, longitudinal dynamics among low abundance taxa can be masked in standard taxa plots and may explain why observed within-subject differences in bacterial community composition rarely track with disease symptoms^9,16,23^. While we cannot rule out that some AHA-negative cells are a result of impaired AHA uptake or instrument detection limits, our data suggest that dead and/or dormant biomass comprise a substantial proportion of sequence reads generated via 16 S rRNA gene sequencing of CF sputum. We hypothesize that low abundance organisms represent keystone members of the lung microbiota, whose activity dynamics are determinants of acute inflammation, either by directly impacting the host, or indirectly through modulating the growth and virulence of higher abundance pathogens.

BONCAT imaging of sputum and fold-change plots between sort-positive and sort-negative fractions demonstrated both population-wide and taxon-specific translational heterogeneity. This spectrum of metabolic states may confer a significant advantage for bacteria and optimize their fitness in the complex environment of the CF lung. In vivo, airway microbiota face a dynamic milieu shaped by microbial competitors, antimicrobials, the host immune response, nutrient limitation, and other chemical stimuli that can be unfavorable to growth. Under these conditions, adopting a bet-hedging strategy in which only a subpopulation of cells is active may ensure that a given bacterial species is prepared to contend with environmental stress^56^. In addition, the transition between translationally active and dormant states may help to explain the periodicity of PEx, faced with a favorable growth environment, more cells of a given taxon (or taxa) may be induced into active growth and elicit a heightened host response.

The balance between growth states may also be a critical determinant of host response to therapy. By adopting a persister-like strategy in which reduced cellular activity confers a temporary multidrug-resistant phenotype, a dormant subpopulation could ensure persistence during an antibiotic challenge. Once antibiotic-selective pressure is relieved, antimicrobial tolerant populations may emerge. This heterogeneity may also help explain instances in which a subject’s clinical response is not predicted by the in vitro drug susceptibility of a given pathogen. We posit that clinical sensitivity panels are poorly predictive of antibiotic efficacy because, among other limitations, they do not account for the heterogeneous translational activity described here.

While active cells are likely more responsible for pathogenesis, inactive cells (Cy5−) are also of importance to CF lung disease as bacteria do not necessarily have to be translationally active to influence their greater community. For example, it is known that largely dormant populations can drive geochemical processes in their growth environment (e.g., mineralizing organic carbon to CO_2_)^57–59^. Translationally inactive cells can also shape their growth environment through nutrient exchange, secretion of virulence factors and small metabolites, electrostatic interactions, and stimulation of the host immune response. Further characterization of activity heterogeneity, the contributions of both active and dormant populations to disease, the frequency of transition between states and the factors that stimulate those transitions will help us to better understand disease dynamics and nature of these.

Though BONCAT represents a useful tool for the study of CF microbiota, we note limitations, several of which have been described previously^39,40,60^. First, bacterial cell sorting by flow cytometry is imperfect, as each species has characteristic sort properties. When defining our gating scheme, Cy5+ and Cy5− gates were conservatively chosen (requiring a gap in between gates) such that the selection of inactive cells in the positive gate was minimized, and vice versa. However, with this gap a subset of the active population is not collected. Similarly, there is a high probability of selecting active cells in the negative gate due to flow migration characteristics (e.g*., F. nucleatum* shifts differently than a much smaller *V. parvula* cell). Finally, bacterial aggregates, in which only some cells are active (see Fig. 3b) could be pulled into the negative gate by the inactive population of that aggregate. We are currently exploring alternative approaches, including optimization of gating strategies, to improve upon the sorting efficiency of BONCAT-labeled cells.

It is also possible that our experimental conditions were selective against certain taxa. As an example, the 3 h AHA incubation is performed under oxic conditions, which may induce an aerobe bloom or inhibit less aero-tolerant bacteria ex vivo. Electrode analyses have shown that steep oxygen gradients are retained in expectorated mucus plugs and are stable over time^50^, but it is notable that after AHA treatment, *Rothia mucilaginosa* (ASV8), *Prevotella salivae* (ASV30), *Veillonella* (ASV13), and other anaerobes were far more prevalent in the negative sort. However, this was not always the case (e.g*., R. dentocariosa* and *S. wiggsiae* increased in subjects 5 and 6) making it difficult to determine whether the observed fold-differences reflect growth constraints during BONCAT labeling or a true slow growth (or dormant) phenotype. Though each bacterium tested in vitro demonstrated the ability to uptake AHA (Fig. 2), it is also expected that each species will incorporate AHA into new proteins at different rates. Similarly, it is possible that catabolism of AHA may skew bacterial composition or induce metabolic changes^61^. Future work will be aimed at optimizing reaction conditions and incubation times to minimize the effect of the experimental approach biasing FACS and sequencing data.

Despite these limitations, BONCAT can be used to extend our understanding of the role of specific microbiota in chronic lung disease. Here we focused on a cross-sectional cohort of stable CF subjects, but the approach can be used to address important questions about microbial community dynamics over time. For example, (i) how do active populations vary with disease state? Future studies will focus on longitudinal analyses of within-subject microbial dynamics and how active species correlate with disease symptoms. By identifying bacterial subpopulations most active either preceding or during an acute disease flare (i.e., PEx), more effective therapeutic strategies are likely to be identified. (ii) Why are only some subjects responsive to antimicrobial therapy? As mentioned above, in vivo drug efficacy is often inconsistent with clinical sensitivity panels. By obtaining sputum and amending small aliquots with different classes of antibiotics, BONCAT analysis of the ensuing changes in bacterial activity can be used to predict how CF subjects might respond to treatment. (iii) How do specific taxa respond to environmental stimuli? It is known that bacteria are dynamically responsive to their growth environment, yet how CF microbiota adapt to perturbations in the sputum milieu is poorly understood. BONCAT characterization of sputum samples amended with specific nutrients or incubation under varying environmental conditions (e.g., low pH) will help to shed light on parameters that constrain or potentiate bacterial growth in vivo. (iv) How is translational activity spatially arranged? With the exception of small bacterial aggregates (Fig. 3), the approach described here offers limited insight on the spatial distribution of bacterial activity. As an alternative to FACS-based sequencing, BONCAT could be combined with species-specific fluorescence in situ hybridization (FISH) probes and histological analysis of sputum (or lung tissue) to visualize spatial relationships between translationally active bacteria^39,40,62^.

In summary, we demonstrate that BONCAT is a powerful tool for the visualization and identification of translationally active bacteria and provides a measure of microbial activity not captured by conventional molecular profiling. Our use of BONCAT lays the foundation for a more detailed understanding of the ecophysiology of CF microbiota and has important implications for the development of new therapeutic strategies and improved clinical outcomes. In addition, the approach is broadly applicable to other airway diseases (e.g., COPD, ventilator associated pneumonias, and sinusitis) where the activity of complex bacterial communities is central to disease states. We are currently using this approach to study microbial community dynamics in a variety of infectious disease contexts.

## Methods

### Bacterial strains and culture conditions

Bacterial strains are listed in Table 1. *Fusobacterium nucleatum, Prevotella melaninogenica, Veillonella parvula*, and *Streptococcus parasanguinis* were derived from the American Tissue Type Collection and obtained from Microbiologics (St. Cloud, MN). *Rothia mucilaginosa* was obtained from the Japan Collection of Microorganisms (Riken, Tokyo). *Staphylococcus aureus, Escherichia coli* and *Pseudomonas aeruginosa* were obtained from D.K. Newman (California Institution of Technology), and *Burkholderia cenocepacia* was obtained from C.H. Mohr (University of Minnesota). *Achromobacter xylosoxidans* and *Stenotrophomonas maltophilia* were isolated from individuals undergoing treatment at the UMN Adult CF Center. Aerobes were maintained on Luria-Bertani (LB) agar, while anaerobes were maintained on Brain-Heart Infusion (BHI) agar supplemented with a 5% vitamin K-hemin solution (Hardy Diagnostics #Z237) in an anaerobic chamber (Coy) under a 90% N_2_/5% CO_2_/5% H_2_ atmosphere. Bacterial growth curves were performed in triplicate in BHI broth containing either 6 mM L-azidohomoalanine (AHA) or 6 mM L-methionine (MET).

**Table 1:** Bacterial strains used in this study.

Bacterial Species	Comment	Source
*Achromobacter xylosoxidans*	CF clinical isolate MN001	75
*Burkholderia cenocepacia*	CF clinical isolate K56-2	76
*Escherichia coli*	UQ950	77
*Fusobacterium nucleatum*	ATCC 25586	ATCC
*Prevotella melaninogenica*	ATCC 25845	ATCC
*Pseudomonas aeruginosa*	Clinical isolate UCBPP-PA14	78
*Rothia mucilaginosa*	JCM 10910	79
*Staphylococcus aureus*	Clinical isolate MN8	80
*Stenotrophomonas maltophilia*	CF clinical isolate CHB83-1	This study
*Streptococcus parasanguinis*	ATCC 15912	ATCC
*Veillonella parvula*	ATCC 10790	ATCC

### Clinical sample collection

Spontaneously expectorated sputum was collected from stable subjects with cystic fibrosis during routine outpatient visits to the Adult CF Center at the University of Minnesota. All subjects provided written informed consent prior to sample collection as approved by the UMN Institutional Review Board (Study #1403M49021). Upon consent, each subject provided a single sample that was collected in a sterile 50 mL conical tube. Cohort data are shown in Supplementary Table 1.

### Bioorthogonal non-canonical amino-acid tagging (BONCAT)

BONCAT labeling was performed as described by Hatzenpichler^40^ with modifications. Briefly, for imaging of lab-grown cultures (see below), *P. aeruginosa, B. cenocepacia, A. xylosoxidans, S. maltophilia, R. mucilaginosa, E. coli,* and *S. aureus* were grown aerobically in LB, while *S. parasanguinis, V. parvula, P. melaninogenica*, and *F. nucleatum* were cultured under anaerobic conditions in BHI broth supplemented with hemin and vitamin K. Cultures were grown overnight and diluted 1/100 in 10 mL of fresh medium. Upon reaching mid-log phase, cultures were supplemented with either 6 mM AHA or 6 mM methionine (MET) and incubated for 3 h at 37 °C. When indicated, an antibiotic cocktail consisting of chloramphenicol (30 µg mL^−1^), tetracycline (200 µg mL^−1^), and tobramycin (10 µg mL^−1^) was added 30 min prior to AHA addition to arrest protein synthesis. After incubation, cultures were pelleted via centrifugation (5 min at 10,000 × *g*), fixed in 4% paraformaldehyde (PFA) for 2 h at 4 °C, resuspended in phosphate buffered saline (PBS, pH 7.4) and stored at 4 °C. All growth curves were performed in triplicate (*n* = 3).

Sputum samples used for imaging were treated with cycloheximide (100 µg mL^−1^) upon expectoration and divided into three equal volumes. Aliquots were supplemented with either AHA (6 mM), methionine (6 mM), or AHA (6 mM) with chloramphenicol/tetracycline/tobramycin as described above, incubated at 37 °C for 3 h, followed by fixation in 4% PFA overnight at 4 °C. Samples collected for flow cytometry were divided into five 300−500 µL aliquots. One control aliquot was immediately frozen at −80 °C and later used for conventional 16 S rRNA gene sequencing. Cycloheximide (100 µg mL^−1^) was added to the remaining four aliquots, three of which were supplemented with AHA (6 mM). One was also supplemented with MET (6 mM), followed by incubation of all samples at 37 °C for 3 h. Labeled samples (and unlabeled controls) were then fixed in 4% PFA for 2 h, pelleted via centrifugation (5 min at 10,000 × *g*), resuspended in PBS, and stored at 4 °C.

### Click chemistry

For each bacterial culture and sputum sample, strain-promoted azide-alkyne cycloaddition (click chemistry)^63^ was also performed as described previously^40^. Briefly, fixed biomass was pelleted, resuspended in freshly prepared 2-chloroacetamide (100 mM) and incubated for 1 h at 46 °C, shaking at 450 r.p.m. in the dark. Cy5-dibenzocyclooctyne (Cy5–DBCO) (Click Chemistry Tools) was then added to a final concentration of 10 µM followed by incubation for 30 min at 46 °C. Samples were washed three times in PBS and further processed for imaging and flow cytometry (see below).

### SDS-PAGE

*P. aeruginosa* was grown to late-exponential phase as described above and supplemented with varying concentrations of AHA (100 µM–1 mM) for 1 h prior to fixation. Similarly, *P. aeruginosa* was grown in the presence of varying ratios of MET:AHA. Bacterial pellets were resuspended in extraction buffer (1% sodium dodecyl sulfate, 50 mM NaCl, 100 mM EDTA, 1 mM MgCl_2_ at pH 8.4) and boiled for 30 min. After boiling, samples underwent click chemistry as described above. A mixture of methanol:chloroform:water (12:3:8) was then added to each sample followed immediately by centrifugation for 5 min at 16,000 × *g*. The water/methanol phase was then carefully removed, and protein recovered from the interface was washed three times in 100% methanol. After the final wash, supernatant was removed and pellets were air dried. Protein was resuspended in 100 μl 1X LDS (lithium dodecyl sulfate) sample buffer and denatured at 70 °C for 10 min. Ten microliter of protein was run on an 8% Bis-Tris gel with MOPS (3-(*N*-morpholino)propanesulfonic acid)-sodium dodecyl sulfate (SDS) running buffer to which sodium bisulfite had been freshly added. Gels were run at 150 V, fixed for 30 min in a 1:2:7 acetate:methanol:water mix, and imaged with a Typhoon FLA 9500 scanner (GE Healthcare) using an excitation wavelength of 635 nm.

### Fluorescence microscopy

BONCAT-labeled bacterial cultures and sputum were spotted on Superfrost Plus microscope slides and counterstained using 1.6 µM STYO64 in PBS. Slides were then washed twice in PBS, mounted using Prolong Diamond Antifade and imaged using an Olympus IX83 microscope with a transmitted Koehler illuminator and a ×60 oil objective lens (NA 1.42). Images were captured on a Hamamatsu ORCA-Flash4.0 V2 digital CMOS camera, and post-acquisition image analysis was performed using cellSens software (v.1.14, Olympus). SYTO64 and Cy5 were visualized using excitation/emission wavelengths of 562 nm/583 nm and 628/640 nm, respectively.

Image analysis was performed using FIJI^64^. Briefly, images were subjected to background subtraction using a rolling ball radius of 150 pixels. Individual cells were identified by adjusting thresholds of SYTO64 images using Huang’s fuzzy thresholding method^65^. Images were also segmented using a watershedding algorithm that assumes each maximum belongs to a discrete particle. The Analyze Particles operation was used to detect and record locations of individual bacterial cells in a given image. For clinical samples, particles were constrained between 100 and 1000 pixels to minimize detection of host cells and sputum debris. Mean pixel intensity at 647 nm (Cy5) was then quantified for each assigned particle. Imaging experiments were performed in triplicate for each bacterial species, and ten images for each sample were captured (*n* > 1000 particles per sample).

### Flow Cytometry

Prior to sorting, Cy5–DBCO-labeled sputum was collected by centrifugation and counterstained with 1.6 µM SYTO9 (Invitrogen) in PBS for 30 min. Sputum samples were also stained with 1 µg ml^−1^ of phycoerythrin (PE) anti-human CD45RO in PBS (BioLegend) for 30 min to stain activated and memory T cells, some B-cell subsets, activated monocytes/macrophages, and granulocytes. All samples were washed in PBS containing 1% BSA and 1 mM EDTA, homogenized using 16- and 22-gauge needles and filtered through a 40 μm cell strainer. To separate AHA + and AHA- bacterial populations, clinical samples were analyzed and sorted on a FACSAriaIIu Cell Sorter (Beckton Dickinson) with a 70μm nozzle at 70 psi. Contaminating human leukocytes staining positive for PE anti-human CD45RO were excluded from bacterial populations of interest in the initial sorting gate (Supplementary Fig. 9). An AHA- control was then matched to each sample to determine the level of non-specific Cy5–DBCO binding and was used to establish Cy5+ (i.e., active) and Cy5− (i.e., inactive) sorting gates. Forward scatter and side scatter gates were then applied to remove large particulates and debris, and liberal doublet discrimination was used to minimize loss of bacterial aggregates. Collected samples were stored at 4 °C and processed within 24 h. FlowJo software (v.10.5.0) was used for data analysis and presentation.

Cy5+ and Cy5− sorted populations were assessed for post-sort purity by flow cytometry, while collected fractions were visualized by anti-Cy5 immunostaining. To do so, BONCAT-labeled sputum samples were spread across Superfrost Plus microscope slides using a sterile pipette tip and allowed to air dry for 30 min. Slides were washed 3X in PBS and blocked using 1% goat serum in PBS for 1 h, followed by treatment with an anti-Cy5 monoclonal antibody (C1117, Sigma–Aldrich) (1:100 dilution) in incubation buffer (1% goat serum, 0.3% Triton X100 and 10 mg mL^−1^ bovine serum albumin) overnight at 4 °C. Slides were washed 3×, and incubated with Cy3 goat anti-mouse secondary antibody (1:250) in incubation buffer for 45 min. Slides were washed 2×, counterstained using 0.1% Hoescht in PBS and mounted using Prolong Diamond Antifade. Slides were imaged as described above.

### DNA extraction

Genomic DNA (gDNA) was extracted using a modified phenol-chloroform method previously described^66^. Briefly, FACS-sorted samples were collected onto 0.22 µm polycarbonate membranes (EMD Millipore), which were then transferred to 1 mL of TENS buffer (50 mM Tris-HCl [pH 8.0], 20 mM EDTA, 100 mM NaCl, 1% SDS) containing lysozyme (0.2 mg mL^−1^) and lysostaphin (0.02 µg mL^−1^) and incubated at 37 °C for 30 min. Sodium dodecyl sulfate (SDS) and proteinase K were added to final concentrations of 1% and 1.2 mg mL^−1^, respectively, and samples were incubated overnight at 55 °C. Enzymes were deactivated by incubating samples at 90 °C for 30 min, and sample liquid (including membrane) was transferred to a 5 mL conical tube containing an equal volume of phenol:chloroform:isoamyl alcohol (P:C:I, 25:24:1, pH 7.9), which dissolved the membrane. The resulting sample was then split into two Lysing Matrix E tubes (MP Biomedicals) and processed twice by bead beating for 30 seconds. Contents of both tubes were recombined and centrifuged at 3200 × *g* for 20 min. The aqueous layer was transferred to a new tube and P:C:I extraction was repeated, followed by a chloroform:isoamyl alcohol (24:1) extraction. A 1/10th volume of sodium acetate (3 M, pH 5.2) was then added and nucleic acid was precipitated using one volume of isopropanol followed by centrifugation at 21,130 × *g* for 20 min. Supernatant was removed, the pellet was washed with 80% ethanol, and centrifuged at 21,130 × *g* for 10 min. Finally, the gDNA pellet was air dried, resuspended in 10 mM Tris buffer (pH 8.0), and stored at −80 °C until sequencing.

### DNA sequencing and analysis

gDNA derived from sputum samples was submitted to the University of Minnesota Genomics Center (UMGC) for 16 S rRNA gene library preparation using a two-step PCR protocol^67^. The V4 variable region was amplified using V4_515F and V4_806R primers with common adapter sequences (5’-TCGTCGGCAGCGTCAGATGTGTATAAGAGACAGGTGCCAGCMGCCGCGGTAA-3’, 5’-GTCTCGTGGGCTCGGAGATGTGTATAAGAGACAGGGACTACHVGGGTWTCTAAT-3’), followed by the addition of dual indices and Illumina flow cell adaptors in a secondary amplification using primers 5’-AATGATACGGCGACCACCGAGATCTACACXXXXXXXXTCGTCGGCAGCGTC-3’ and 5’-CAAGCAGAAGACGGCATACGAGATXXXXXXXXGTCTCGTGGGCTCGG-3’. Amplicons were sequenced on an Illumina MiSeq using TruSeq (v.3) 2 × 300 paired-end technology. FACS sheath fluid and DNA extraction reagent control samples were also submitted for sequencing. These control samples did not pass quality control steps due to DNA content below detection thresholds but were incorporated into downstream analyses. An average of 67,793 sequences per sample were obtained. Sequence data are available at NCBI sequence read archive under Bioproject ID PRJNA604587.

Sequence quality was assessed using the DADA2 R package (v.1.2.1)^55^. Cutadapt^68^ was used to remove primer and Illumina adapter sequences, with size filtering set to a minimum and maximum of 215 bp and 285 bp, respectively. DADA2 functions were used to trim and filter sequences, model and correct Illumina sequence errors, align paired-end sequences, and filter chimeric reads. Specifically, forward and reverse sequences were trimmed to 250 bp and 200 bp, respectively, and a post-trimming minimum length filter of 175 bp was applied. All other DADA2 pipeline parameters were run using default options. Resulting amplicon sequence variants (ASVs) were assigned taxonomy using RDP classifier^69^ and the SILVA SSU database (Release 132, December 2017)^70,71^. Species-level taxonomy was assigned using the DADA2 addSpecies function only if an ASV unambiguously matched a sequence in the SILVA-132 database. A phylogenetic tree was approximated using the phangorn R package^72^ and sequences were aligned using DECIPHER. The phangorn package was then used to construct a neighbor-joining tree, which was then used to fit a GTR + G + I maximum likelihood tree.

The Decontam package (v.1.2.0)^73^ was used to reproducibly filter out contaminant sequences. The function isContaminant was used with method = “either” and a probability threshold set to 0.5. Frequency was determined from 16 S qPCR data obtained from UMGC. A total of 28 taxa were removed from the dataset based on frequency and prevalence in the sample when compared with DNA extraction control. An average of 40,773 sequences were recovered from DADA2/Decontam analysis corresponding to 357 ASVs. 79.55% of ASVs were assigned to the genus level, and 22.97% had an unambiguous species assignment.

ASV count data, taxonomic assignment, and the phylogenetic tree were used within the analysis framework of the Phyloseq R package (v.1.26.0)^74–80^. ASVs were filtered when they did not belong to the domain Bacteria, or when not assigned taxonomy at the phylum level. Phyla that had low prevalence and abundance (including Acidobacteria, Chloroflexi, Dependentiae, Planctomycetes, and Synergistetes) were removed from the dataset, as were singleton ASVs or those that did not belong to the original or input samples. Finally, ASVs at a relative abundance below 0.001 (0.1%) were removed. After filtering there remained 45 unique taxonomic assignments with 22 assigned at the species level. Fold-change in relative abundance for each ASV were calculated between sort input and sort-positive fractions for each study subject. For all figures, a specific epithet was used when assigned exactly from the SILVA database

### Reporting summary

Further information on research design is available in the Nature Research Reporting Summary linked to this article.

## Supplementary information

Supplementary Information

Reporting Summary

## Data Availability

Raw 16 S rRNA gene sequence data (Fig. 5 and Supplementary Figs. 8, 11-13) that support the findings of this study were deposited and are available as fastq files in the NCBI sequence read archive under Bioproject ID PRJNA604587. Source data and full gel scans underlying Figs. 1, 3, 5, and Supplementary Figs. 1, 2, 4, 6, 12, and 13 are provided in the Source Data file.

## Source Data

Source Data

## Code availability

Previously published software packages and versions used to analyze 16 S rRNA sequence data are cited in the methods above. The custom R function used in sequence analysis is available on Github.
