# Supplementary material for: Bioorthogonal non-canonical amino acid tagging reveals translationally active subpopulations of the cystic fibrosis lung microbiota
Source: Nat Commun. 2020 May 8;11:2287. doi: 10.1038/s41467-020-16163-2 (PMC7210995; doi:10.1038/s41467-020-16163-2)
Supplement: Supplementary file 1 — Supplementary Information [file 41467_2020_16163_MOESM1_ESM.pdf]

Bioorthogonal non-canonical amino acid tagging reveals translationally active subpopulations of the cystic fibrosis lung microbiota.

Valentini *et al.*

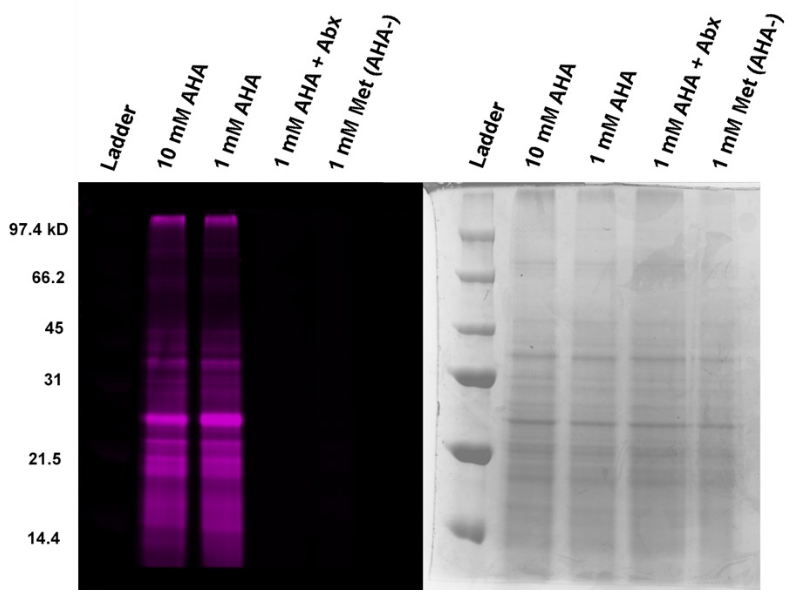

Supplementary Figure 1. BONCAT labeling of *P. aeruginosa* is specific for AHA and translational activity. SDS-PAGE visualization of BONCAT labeling of laboratory cultures of *P. aeruginosa* PA14. Labeling is specific for AHA and inhibited by antibiotics (Abx = chloramphenicol, tetracycline, tobramycin). Full gel scans are provided as a Source Data file.

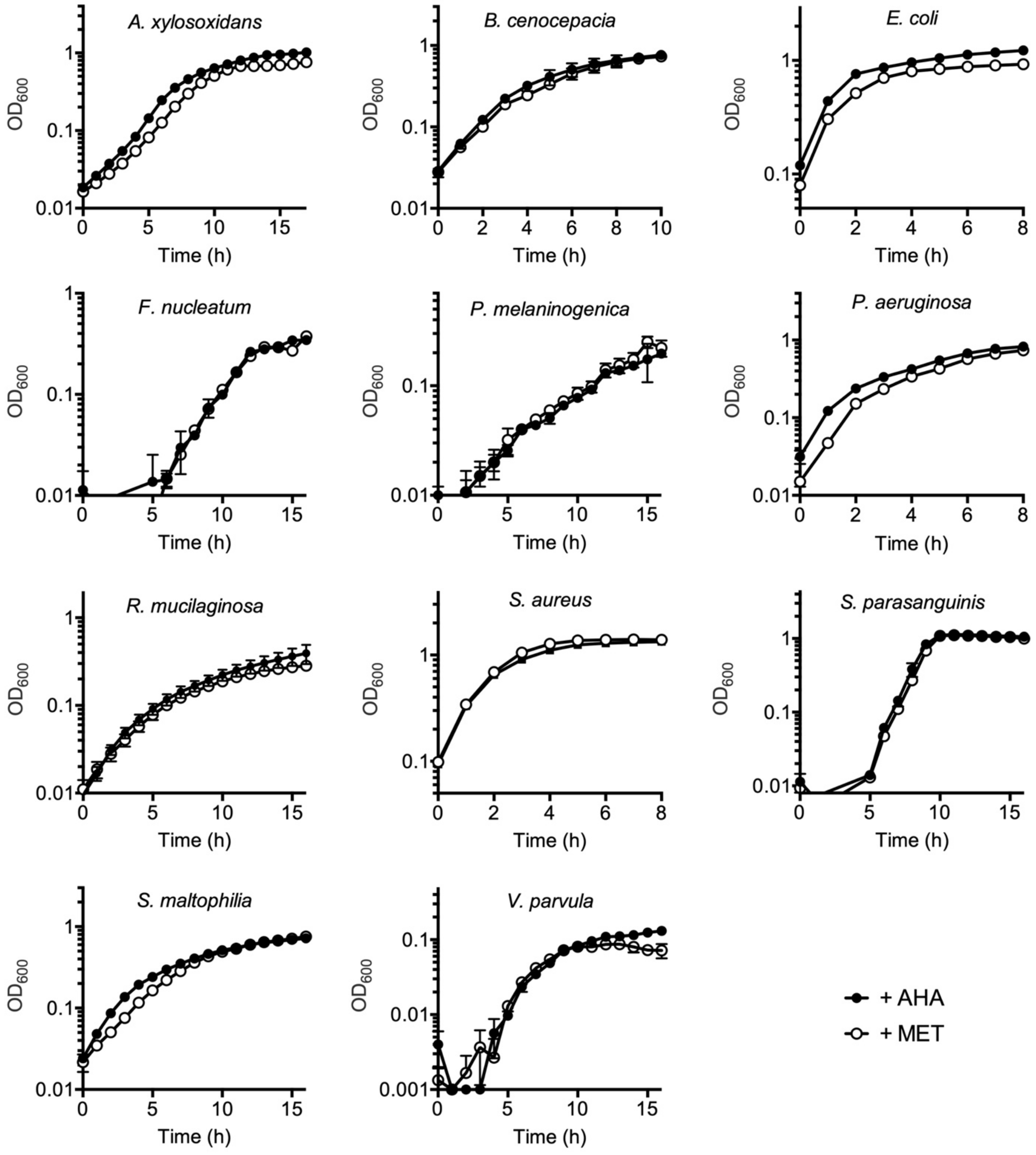

Supplementary Figure 2. AHA-incubation has negligible effect on bacterial growth. Growth curves were performed in triplicate for each species under each growth condition (6mM L-azidohomoalanine (AHA) or 6mM methionine (MET)). Error bars represent standard deviation of the mean of three biological replicates. Source data are provided as a Source Data file.

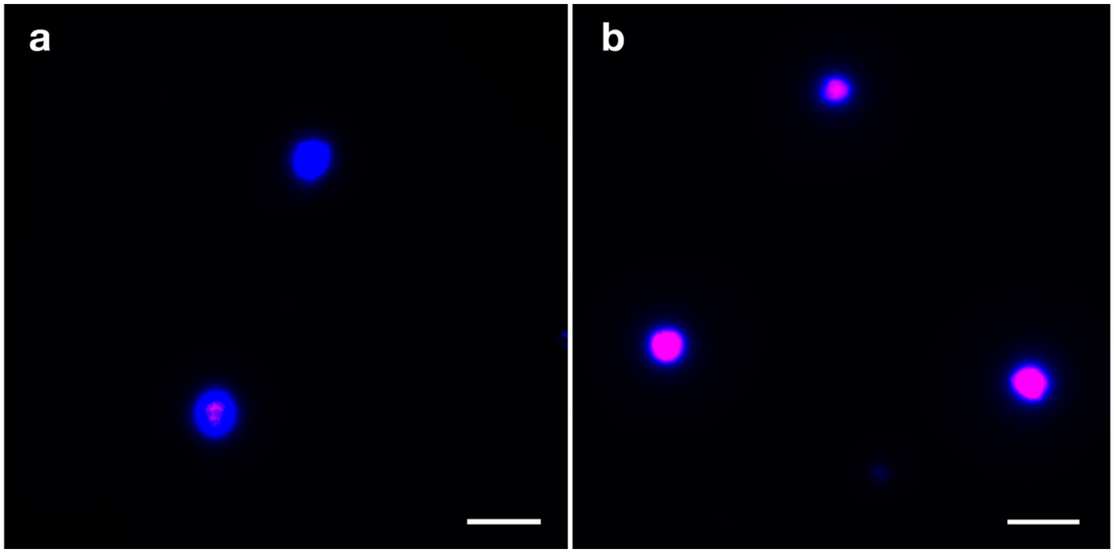

Supplementary Figure 3. Cycloheximide Inhibition of host cell BONCAT labeling. RAW 264.7 macrophages (Sigma Aldrich 91062702) were cultured in T25 flasks containing high glucose Dulbecco's Modified Eagle's Medium supplemented with GlutaMAX and pyruvate (Thermo, 10569044). Cycloheximide was added to one flask at a final concentration of 100 μg/mL, and another was left untreated. AHA was then added to a final concentration of 6mM to both flasks and incubated for 3h. Cells were detached using 0.25% trypsin solution in EDTA (Sigma T4049) and fixed in 1mL of 4% PFA in PBS for 2 h prior to imaging. Imaging was performed as described in the main text. Representative images of two biologically independent experiments are shown. **(a)** BONCAT labeling of macrophages to which cycloheximide was added were inhibited relative to **(b)** untreated (i.e. AHA+ cells). Bar = 20 μM.

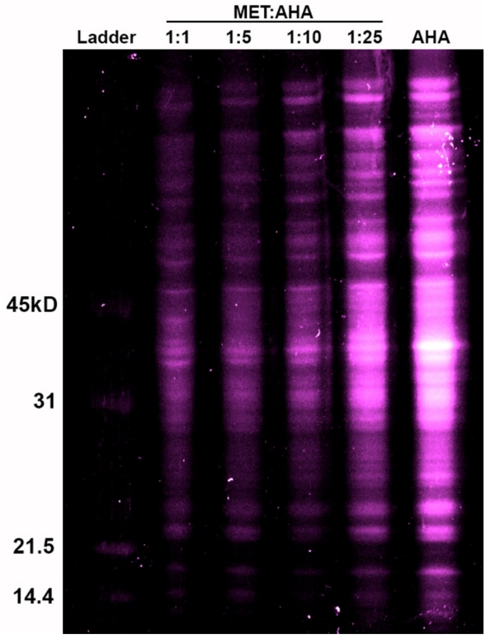

Supplementary Figure 4. SDS-PAGE determination of MET:AHA ratio required for BONCAT labeling of *P. aeruginosa*. Cells were grown in varying concentrations of MET:AHA prior to labeling with Cy5-DBCO. Based on these profiles, a 1:10 ratio was selected for labeling of *in vitro* bacterial cultures and expectorated sputum samples. These data are representative of three independent experiments. Full gel scans are provided as a Source Data file.

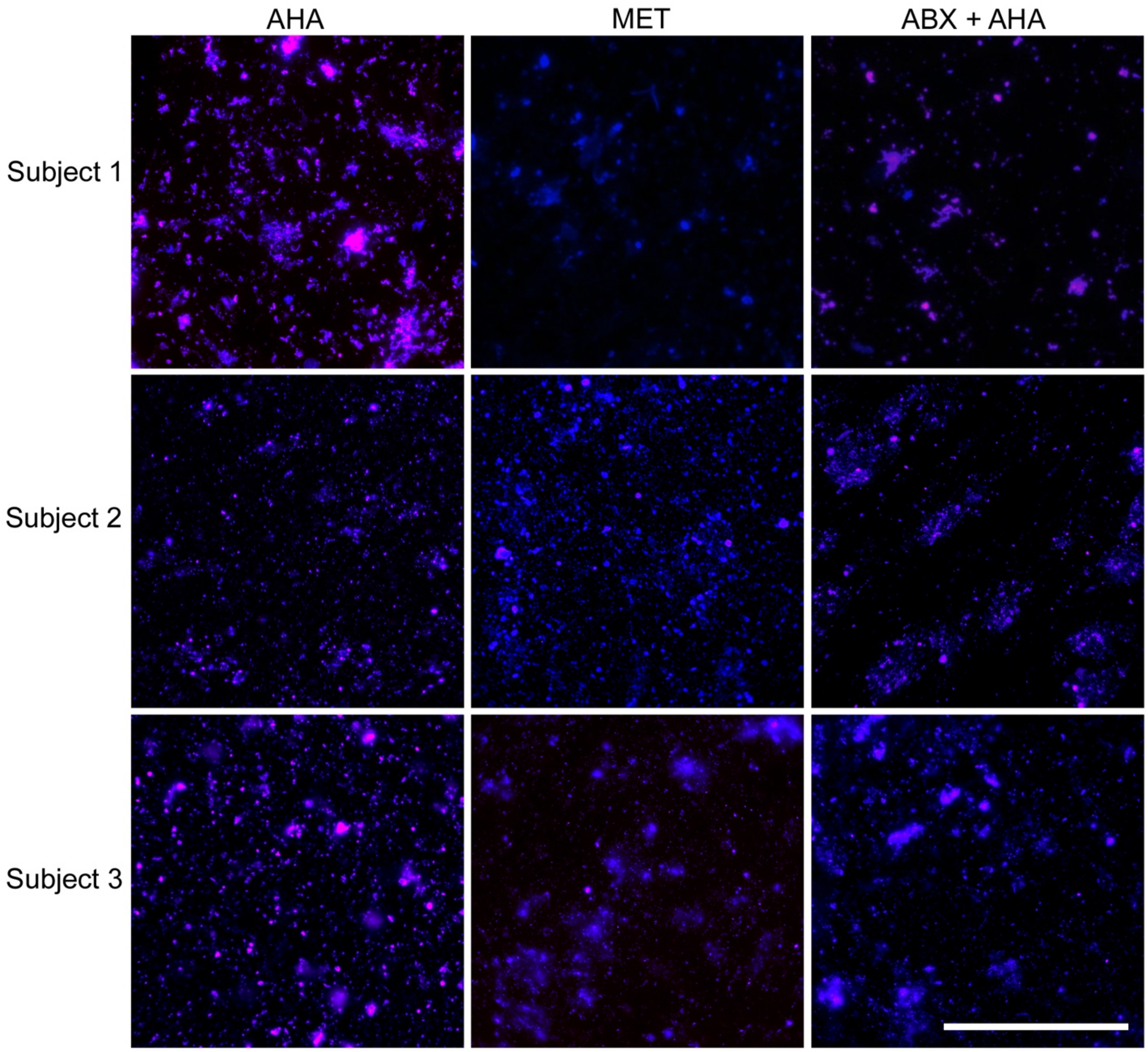

Supplementary Figure 5. BONCAT labeling of sputum microbiota is specific for AHA. Expectorated sputum samples were supplemented with 6mM AHA and incubated for 3h prior to Cy5-DBCO labeling (Cy5; magenta) and counterstaining (SYTO64; blue). Negligible background fluorescence was observed in paired sputum samples incubated with methionine (MET). Incubation with antibiotics prior to AHA labeling (ABX+AHA) resulted in a moderate decrease in fluorescence that may reflect antimicrobial tolerance among airway microbiota. Bar = 100 µm. Abx = chloramphenicol, tetracycline, tobramycin. Data are representative of ten images from each patient sample.

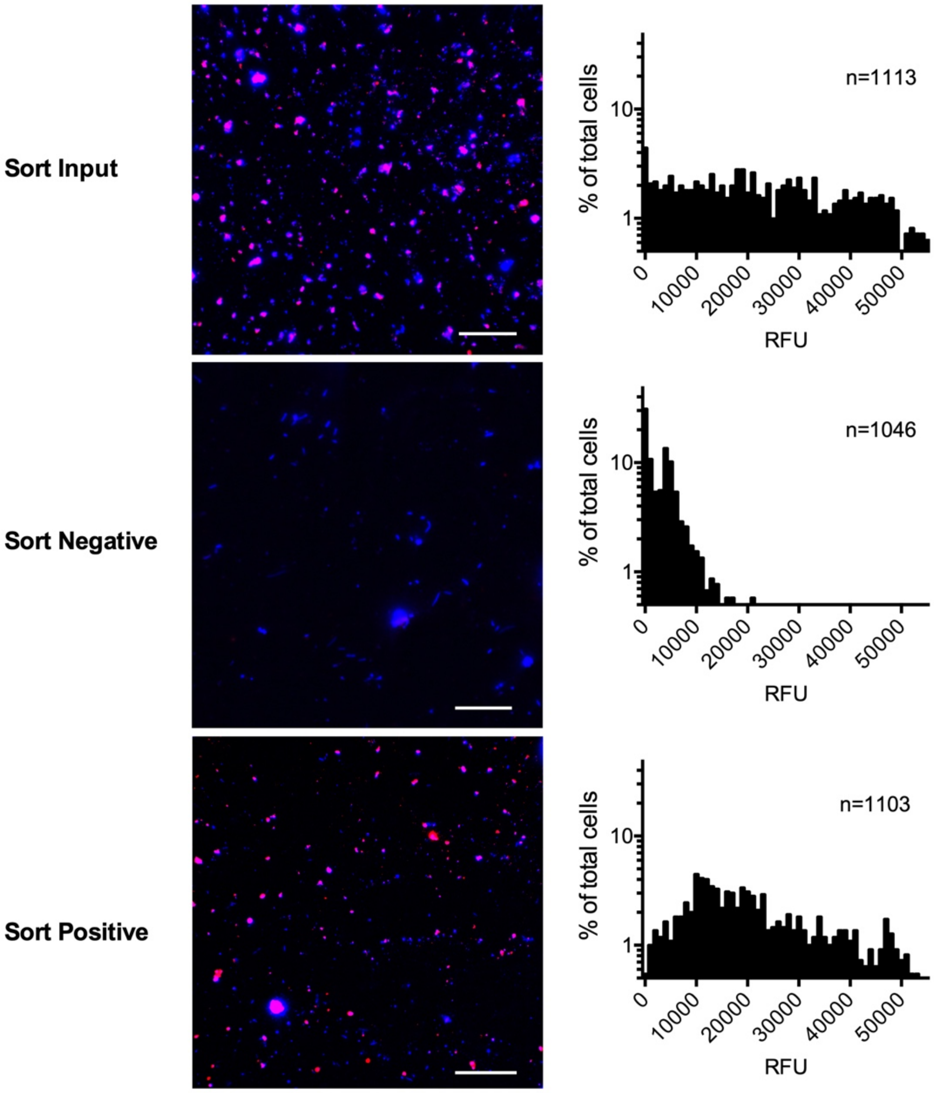

Supplementary Figure 6. Validation of BONCAT-FACS. During sorting, the Cy5 label is subject to photobleaching; we therefore used immunostaining (Cy3 anti-Cy5 antibody), fluorescence microscopy and quantitative image analysis in FIJI to validate FACS sort integrity. Pixel intensity was calculated as described in Methods. Anti-Cy5 labeling of sort input, sort negative and sort positive fractions demonstrates that BONCAT-FACS effectively removes host cells and translationally inactive bacteria from downstream 16S rRNA gene sequencing analysis. As expected, more cells in the positive fraction were anti-Cy5 reactive, confirming AHA uptake and translational activity among this bacterial subpopulation. However, some collected cells appeared unlabeled by the Cy3-labeled antibody. If we assume a mean pixel intensity of 10,000 as the cutoff between AHA-/AHA+ cells, these data suggest false negative and false positive sort rates of 6.8% and 12.0%, respectively. Bar = 10 μm. Source data are provided as a Source Data file.

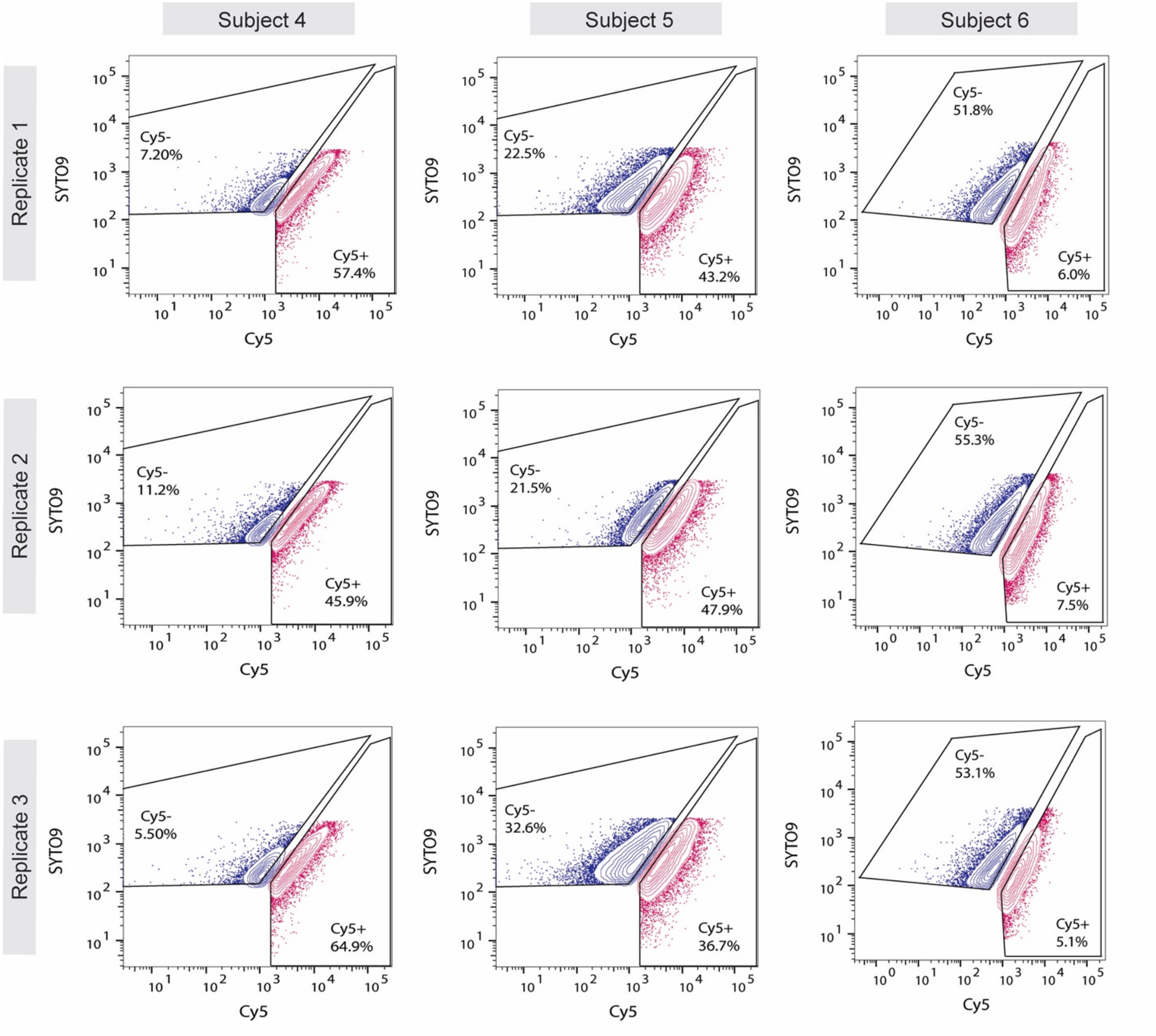

Supplementary Figure 7. FACS of BONCAT-labeled sputum reveals consistency of Cy5- and Cy5+ subpopulations between replicates. Percentages shown reflect % of parent population post-CD45RO gating.

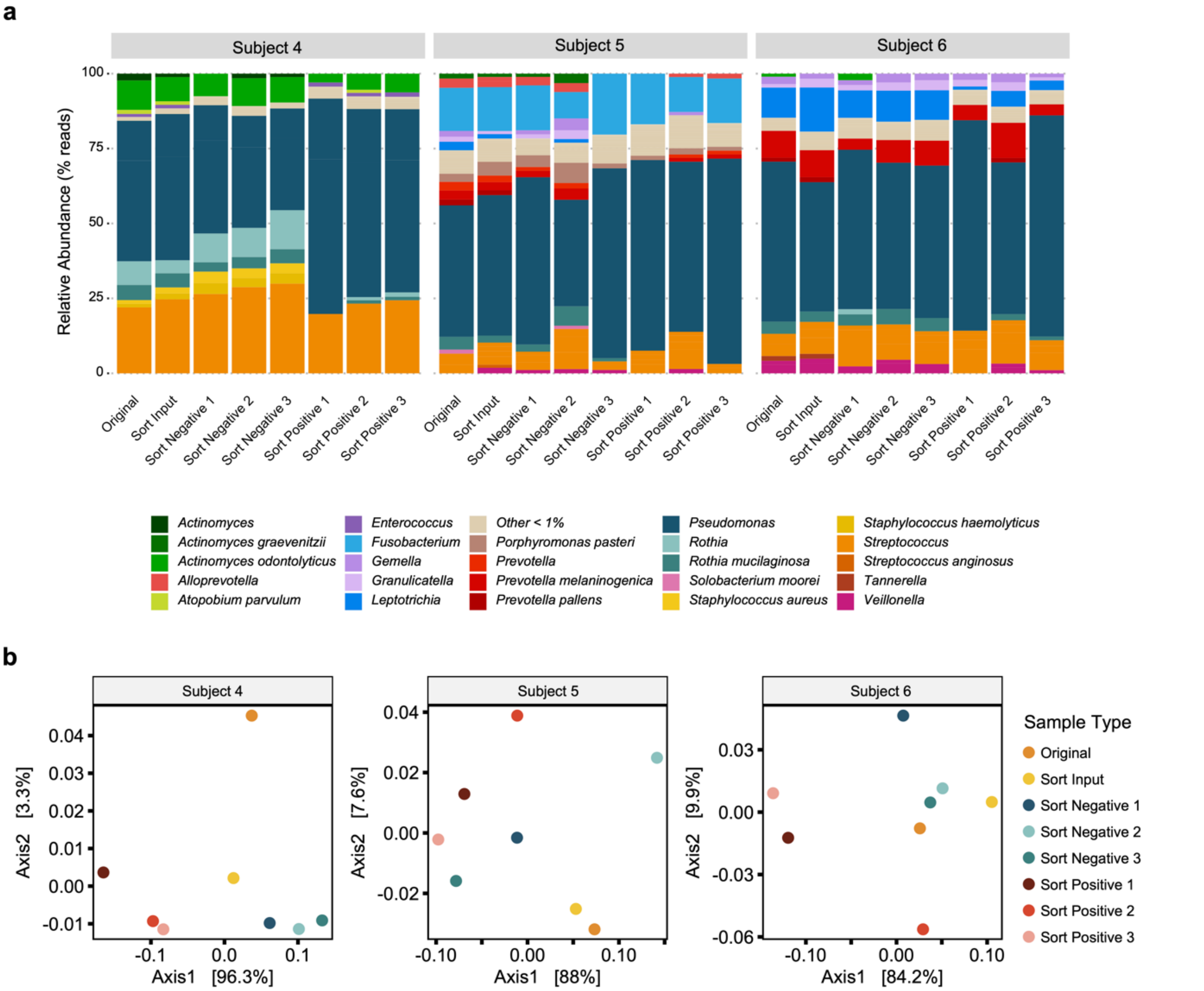

Supplementary Figure 8. 16s rRNA gene sequencing reveals concordance of sort positive and sort negative replicates. **(a)** Stacked bar graphs of taxa relative abundance including all samples in analysis (triplicate positive and negative sort). **(b)** Double Principle Coordinate Analysis (DPCoA) shows grouping of samples by sample type.

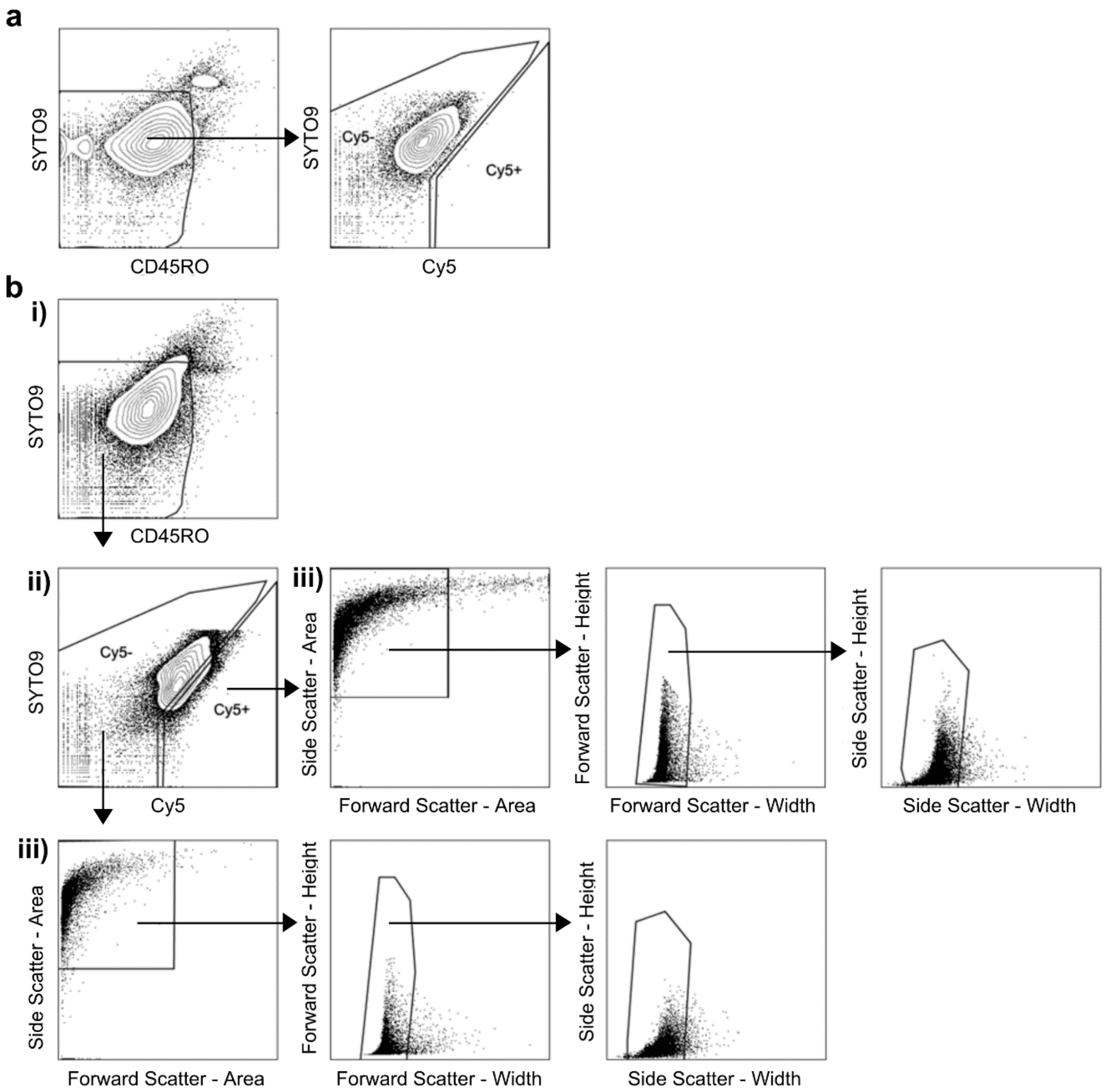

Supplementary Figure 9. Representative gating scheme for AHA+ and AHA- populations. **(a)** AHA- control samples were first gated based on PE anti-human CD45RO staining to remove human leukocytes. Next, the negative control was used to measure background Cy5 fluorescence to define Cy5+ (*i.e.* active) and Cy5- (*i.e.* inactive) sorting gates. **(b)** shows a subject-matched AHA+ sample and the gating used to isolate populations of interest. The gating strategy involved, **i)** excluding human leukocytes using PE anti-human CD45RO, **ii)** using Cy5+ and Cy5- gates based on the subject-matched AHA- control, **iii)** creating forward scatter and side scatter plots to remove large, complex particulates and debris, and liberal doublet discrimination to minimize the loss of bacterial aggregates. The gating shown in panel ii corresponds to FACS data panels shown in Figures 5, S7, and S10.

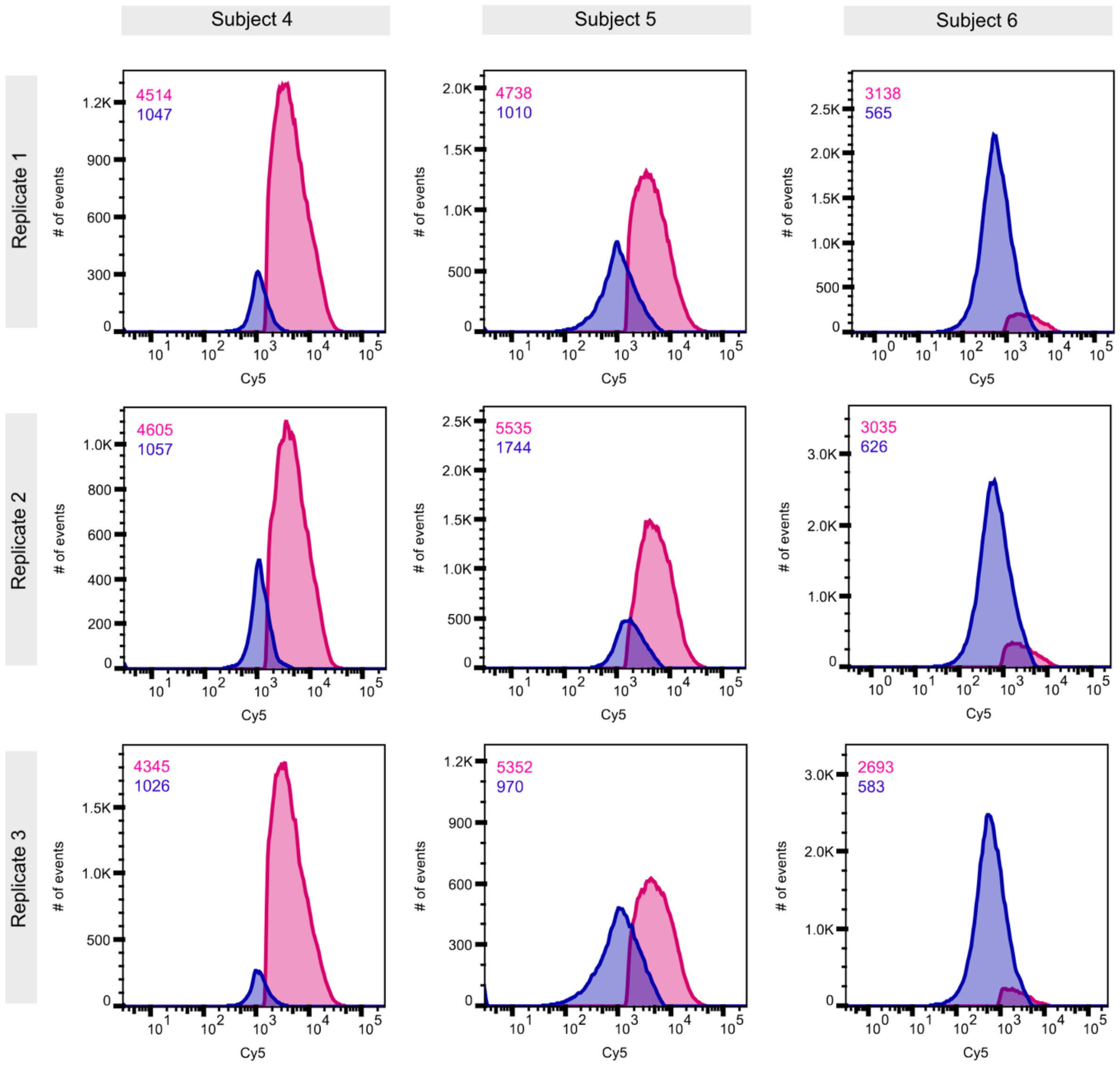

Supplementary Figure 10. Confirmation of BONCAT Labeling. Flow cytometry histograms represent the number of events (cell counts) versus the log fluorescent intensity of Cy5 (blue = Cy5- population, magenta = Cy5+ population). Numbers in the upper left-hand corner represent the geometric means of Cy5 intensity. The Cy5+ population exhibited a higher geometric mean of fluorescent intensity validating AHA uptake and BONCAT labeling of the active subpopulation from CF sputum samples.

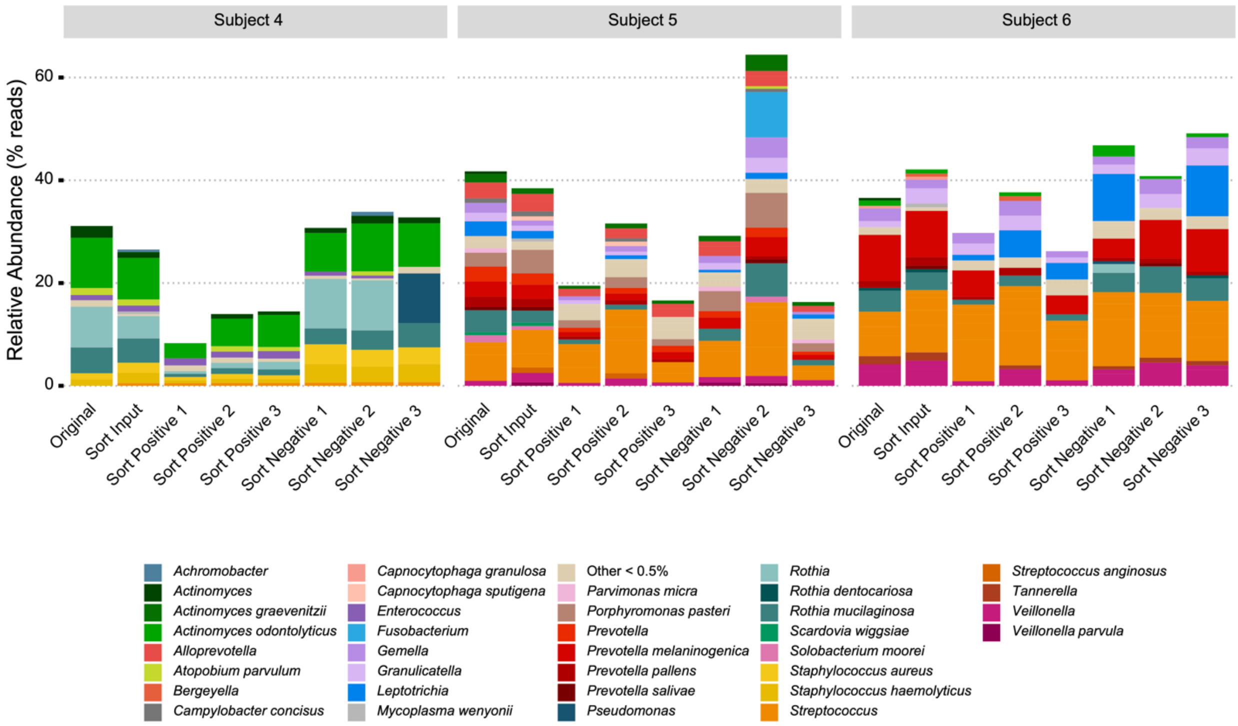

Supplementary Figure 11. Low abundance community members are translationally active. Bacterial community membership of taxa with relative abundances less than 10% exhibit notable differences between “sort positive” community composition (i.e. translational activity) relative to the “original” fraction.

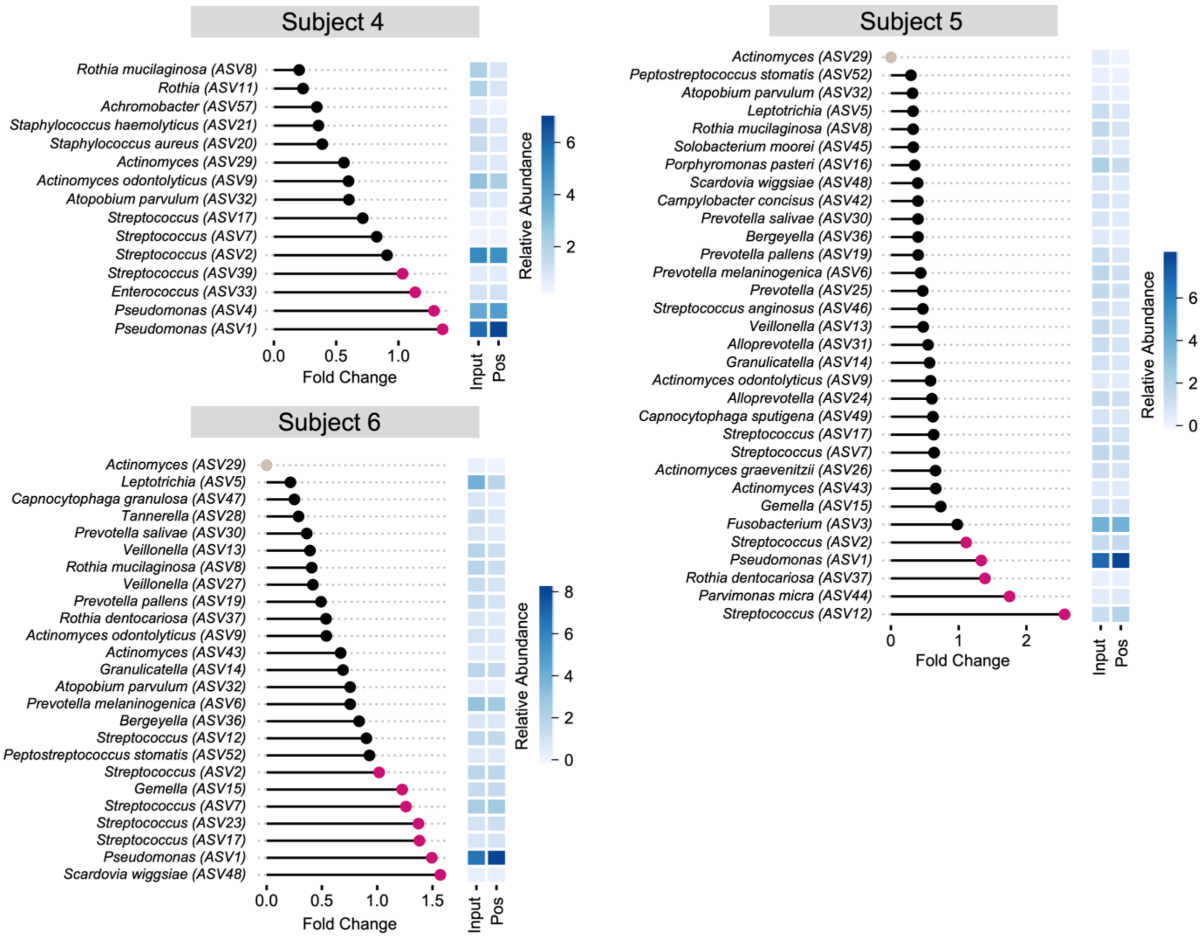

Supplementary Figure 12. Fold difference in taxon relative abundance between “sort input” and “sort positive” fractions. Pink and black markers indicate taxa that were higher in relative abundance in the positive and input fractions, respectively. Source data are provided as a Source Data file.

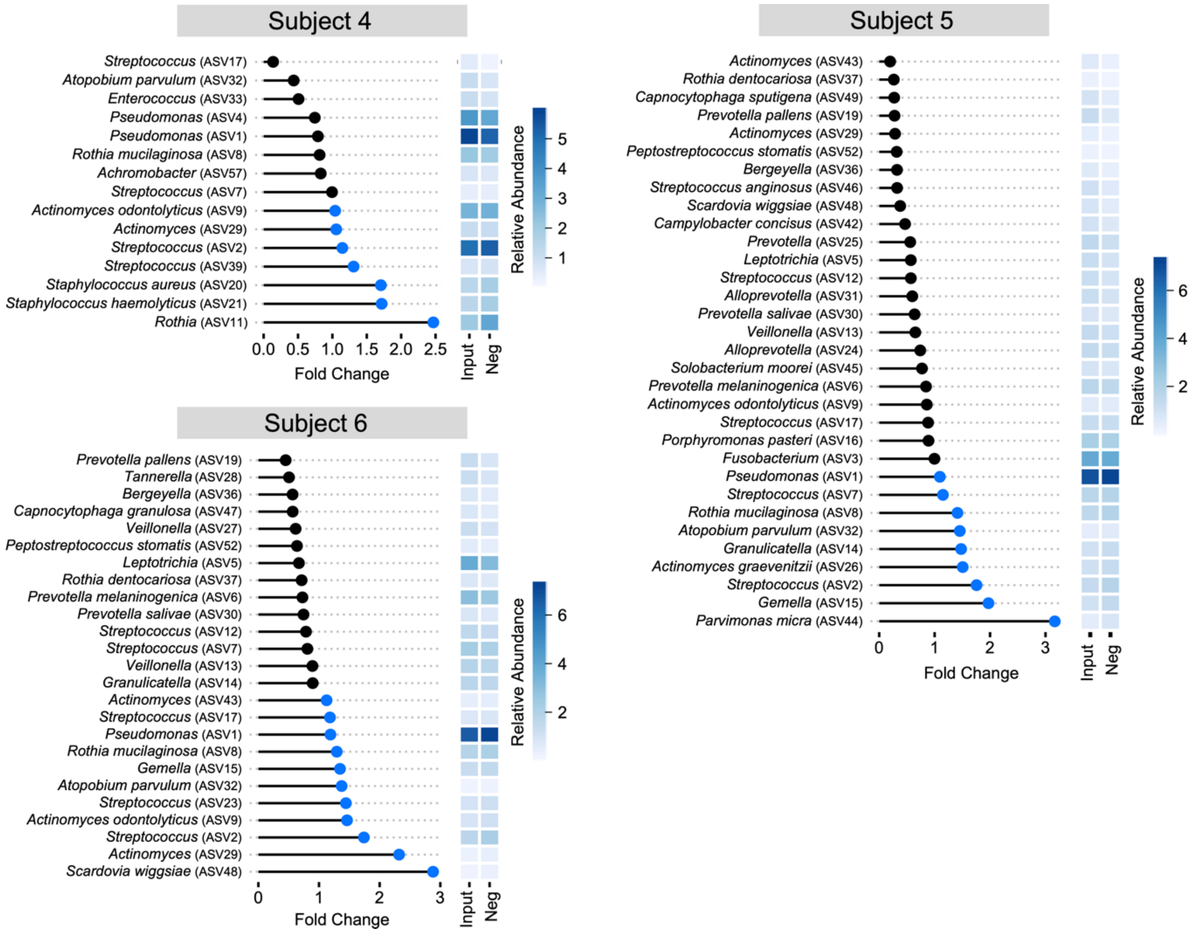

Supplementary Figure 13. Fold difference in taxon relative abundance between “sort input” and “sort negative” fractions. Black and blue markers indicate taxa that were higher in relative abundance in the input and negative fractions, respectively. Source data are provided as a Source Data file.

Supplementary Table 1. Cohort clinical data

Subject	Age	Sex	FEV1%	CFTR genotype	Bacterial Cultures	Current Antibiotics
1	23	F	38	∆F508/∆F508	*A. xylosoxidans*	azithromycin, colistin, doxycycline
2	38	M	31	∆F508/∆F508	*A. xylosoxidans,*<br>*B. cepacia complex,*<br>*P. aeruginosa*	azithromycin, aztreonam, doxycycline, meropenem, sulfamethoxazole-trimethoprim, zosyn
3	23	M	56	∆F508/∆I507	*P. aeruginosa*,<br>*S. aureus*	azithromycin, aztreonam, tobramycin
4	26	F	42	∆F508/N1303K	*S. aureus*<br>*P. aeruginosa*<br>*A. xylosoxidans*	azithromycin, colistin, gentamicin, sulfamethoxazole-trimethoprim
5	40	F	39	1898+1g>a/3849+10kbC>t	*P. aeruginosa*	Azithromycin, aztreonam, ciprofloxacin
6	42	M	38	F508del/621+1G->T	*P. aeruginosa*	Azithromycin, aztreonam, doxycycline

Supplementary Table 2. BONCAT-FACS summary

Subject	Replicate	# of Cy5- events collected	# of Cy5+ events collected	% of Parent population[a] in Cy5- gate	% of Parent population[a] in Cy5+ gate	Geometric mean of fluorescent intensity (Cy5)[b] in Cy5- gate	Geometric mean of fluorescent intensity (Cy5)[b] in Cy5+ gate
4	1	1,632,712	4,000,000	7.2	57.4	1047	4514
	2	3,061,496	4,010,359	11.2	45.9	1057	4605
	3	1,329,894	4,000,000	5.5	64.9	1026	4345
5	1	1,983,509	4,001,293	22.5	43.2	1010	4738
	2	2,770,068	4,003,957	21.5	47.9	1744	5535
	3	4,000,000	4,003,615	32.6	36.7	970	5352
6	1	4,000,000	2,144,345	51.8	6.0	565	3138
	2	4,000,000	3,845,123	55.3	7.5	626	3035
	3	4,000,000	1,717,276	53.1	5.1	583	2693

[a] Percentages reflect % of parent population post-CD45RO gating. Remaining counts fell outside Cy5-/Cy5+ gates.
[b] Histograms are shown in Supplementary Figure 8.
.
