# Supplementary material for: Bioorthogonal non-canonical amino acid tagging reveals translationally active subpopulations of the cystic fibrosis lung microbiota
Source: Nat Commun. 2020 May 8;11:2287. doi: 10.1038/s41467-020-16163-2 (PMC7210995; doi:10.1038/s41467-020-16163-2)
Supplement: Supplementary file 2 — Reporting Summary [file 41467_2020_16163_MOESM2_ESM.pdf]

nature research

Corresponding author(s): RC Hunter

Last updated by author(s): Apr 9, 2020

Reporting Summary

Nature Research wishes to improve the reproducibility of the work that we publish. This form provides structure for consistency and transparency in reporting. For further information on Nature Research policies, see Authors & Referees and the Editorial Policy Checklist .

Statistics

For all statistical analyses, confirm that the following items are present in the figure legend, table legend, main text, or Methods section.

n/a	Confirmed	
☐	☒	The exact sample size (*n*) for each experimental group/condition, given as a discrete number and unit of measurement
☐	☒	A statement on whether measurements were taken from distinct samples or whether the same sample was measured repeatedly
☐	☒	The statistical test(s) used AND whether they are one- or two-sided<br>*Only common tests should be described solely by name; describe more complex techniques in the Methods section.*
☒	☐	A description of all covariates tested
☒	☐	A description of any assumptions or corrections, such as tests of normality and adjustment for multiple comparisons
☐	☒	A full description of the statistical parameters including central tendency (e.g. means) or other basic estimates (e.g. regression coefficient) AND variation (e.g. standard deviation) or associated estimates of uncertainty (e.g. confidence intervals)
☒	☐	For null hypothesis testing, the test statistic (e.g. *F*, *t*, *r*) with confidence intervals, effect sizes, degrees of freedom and *P* value noted<br>*Give P values as exact values whenever suitable.*
☒	☐	For Bayesian analysis, information on the choice of priors and Markov chain Monte Carlo settings
☒	☐	For hierarchical and complex designs, identification of the appropriate level for tests and full reporting of outcomes
☒	☐	Estimates of effect sizes (e.g. Cohen's *d*, Pearson's *r*), indicating how they were calculated

Our web collection on statistics for biologists contains articles on many of the points above.

Software and code

Policy information about availability of computer code

Data collection	Flow cytometry data was collected using a BD FACS Aria ii cytometer with BD-FACSDiva software (v8.0.1). Sequencing data were collected on an Illumina MiSeq instrument with Illumina software. Imaging data were collected on an Olympus IX83 inverted microscope with a Hamamatsu ORCA-Flash4.0 v2 camera. Image acquisition and post-acquisition image analysis was performed using CellSens software (v1.14, Olympus)
Data analysis	Image analysis was also performed using FIJI (v.2.0). Flow cytometry data was analyzed using FlowJo software (v.10.5). All analyses of Illumina MiSeq generated 16S rRNA gene sequences was done using the R language within R Studio software (v.1.2.1335). Sequences were trimmed and filtered for quality using cutadapt (v.2.8). The DADA2 (v.1.14) package was used to model sequencing errors and determine amplicon sequence variants (ASVs) from raw sequencing reads. Decontam (v.1.2)was used to filter out contaminant sequences. phangorn (v.2.5.5) was used to approximate a phylogenetic tree. 'Phyloseq' (v.1.30.0) was used for data quality filtering. Taxonomy was assigned using RDP classifier and SILVA SSU database (release 132). A custom R function was written to calculate and plot fold changes in relative abundance (Fig.5, Supp. Fig. 12, 13) All R code used in sequence analysis in this publication is available as a Github repository (https://github.com/hunterlabumn/Valentini_et_al_2020).

For manuscripts utilizing custom algorithms or software that are central to the research but not yet described in published literature, software must be made available to editors/reviewers. We strongly encourage code deposition in a community repository (e.g. GitHub). See the Nature Research guidelines for submitting code & software for further information.

Data

Policy information about availability of data

All manuscripts must include a data availability statement. This statement should provide the following information, where applicable:

- Accession codes, unique identifiers, or web links for publicly available datasets
- A list of figures that have associated raw data
- A description of any restrictions on data availability

Raw 16S rRNA gene sequence data (Fig. 5 and Supplementary Figures 8, 11-13) that support the findings of this study were deposited and are available as fastq files

in the NCBI sequence read archive under Bioproject ID PRJNA604587. Source data and full gel scans underlying Figures 1,3,5, and Supplementary Figures 1,2,4,6,12, and 13 are provided in the Source Data File.

Field-specific reporting

Please select the one below that is the best fit for your research. If you are not sure, read the appropriate sections before making your selection.

- [x] Life sciences
- [ ] Behavioural & social sciences
- [ ] Ecological, evolutionary & environmental sciences

For a reference copy of the document with all sections, see nature.com/documents/nr-reporting-summary-flat.pdf

Life sciences study design

All studies must disclose on these points even when the disclosure is negative.

Sample size

No sample size calculations were performed for this proof-of-concept study. The small sample size (n=3 each for imaging and flow cytometry) was sufficient to demonstrate the applicability and reproducibility of the method across patients.

Data exclusions

No data or subjects were excluded from the analysis.

Replication

All human samples and in vitro experiments were performed and analyzed in triplicate. All attempts at replication were successful.

Randomization

Randomization was not applicable for this proof-of-concept study demonstrating the utility of the method. No comparisons were made between or across samples.

Blinding

Blinding was not applicable for this proof-of-concept study demonstrating the utility of the method. The sources and treatment of all samples was identical.

Reporting for specific materials, systems and methods

We require information from authors about some types of materials, experimental systems and methods used in many studies. Here, indicate whether each material, system or method listed is relevant to your study. If you are not sure if a list item applies to your research, read the appropriate section before selecting a response.

Materials & experimental systems

n/a	Involved in the study	
[ ]	[x]	Antibodies
[ ]	[x]	Eukaryotic cell lines
[x]	[ ]	Palaeontology
[x]	[ ]	Animals and other organisms
[ ]	[x]	Human research participants
[x]	[ ]	Clinical data

Methods

n/a	Involved in the study	
[x]	[ ]	ChIP-seq
[ ]	[x]	Flow cytometry
[x]	[ ]	MRI-based neuroimaging

Antibodies

Antibodies used

Mouse anti-cy5 monoclonal antibody (Sigma-Aldrich, Cat#C1117, Clone: CY5-15, Lot#059M4798V)

IgG (H+L) Cross-Adsorbed Goat anti-Mouse, Cyanine3 (Invitrogen, Cat#A10521, lot#1855013)

PE anti-human CD45RO (BioLegend, Cat#304244, Clone: UCHL1, lot#B276297) (1:200 dilution)

Validation

https://www.sigmaaldrich.com/catalog/CertOfAnalysisPage.do?symbol=C1117&LotNo=059M4798v&brandTest=SIGMA&returnUrl=%2Fproduct%2FSIGMA%2FC1117 (mouse anti-cy5 monoclonal antidosyy certificate of analysis.

https://www.thermofisher.com/document-connect/document-connect.html?url=https%3A%2F%2Fassets.thermofisher.com%2FTFS-Assets%2FLSG%2Fcertificate%2FCertificates%2520of%2520Analysis%2F1855013_A10521.pdf&title=TG90ICMmbmJzcDsxODU1MDEz (cy3 gota antimouse IgG certificate of analysis)

https://www.biolegend.com/Default.aspx?Id=18921 (PE anti0human CD45RO certificate of analysis). Manufacturer quality control tests by immunofluorescent staining with flow cytometry analysis.

Eukaryotic cell lines

Policy information about cell lines

Cell line source(s)	RAW 264.7 murine macrophages (Sigma Aldrich 91062702)
Authentication	Not authenticated
Mycoplasma contamination	Cell lines were not tested for Mycoplasma contamination.
Commonly misidentified lines (See ICLAC register)	No commonly misidentified cell lines were used in the study.

Human research participants

Policy information about studies involving human research participants

Population characteristics	Adult (>18years of age), diagnosed with cystic fibrosis. Three male and three female subjects were recruited.
Recruitment	Patients were recruited during routine outpatient, pulmonary function testing at the Adult CF Center at the University of Minnesota. Any selection bias should not impact the results of the study.
Ethics oversight	UMN Institutional Review Board (Study #1403M49021)

Note that full information on the approval of the study protocol must also be provided in the manuscript.

Flow Cytometry

Plots

Confirm that:

- [x] The axis labels state the marker and fluorochrome used (e.g. CD4-FITC).
- [x] The axis scales are clearly visible. Include numbers along axes only for bottom left plot of group (a 'group' is an analysis of identical markers).
- [x] All plots are contour plots with outliers or pseudocolor plots.
- [x] A numerical value for number of cells or percentage (with statistics) is provided.

Methodology

Sample preparation	Spontaneously expectorated sputum from cystic fibrosis patients were treated with (100ug/ml) cycloheximide and divided four ways. Three aliquots were supplemented with AHA (6mM) and one with methionine (6mM), followed by incubation of all samples at 37C for 3h. Sputum samples were then fixed in 4% PFA for 2h, washed and resuspended in PBS, and stored at 4C. The day before flow cytometry, sputum samples were incubated in 2-chloroacetamide (100mM) for 1h at 46C, shaking at 250rpm in the dark. Cy5-dibenzocyclooctyne (Cy5-DBCO) (Click Chemistry Tools) was then added to a final concentration of 10µM followed by incubation for 30 min at 46°C. Samples were washed three times in PBS and stained with 1.6uM SYTO9 and 1ug/mL PE anti-human CD45RO in PBS for 30min. All sample were then washed in PBS containing 1% BSA and 1mM EDTA, homogenized using 16- and 22-gauge needles and filtered through a 40µm cell strainer.
Instrument	BD FACSAria II P07800142 with a 70um nozzle at 70psi.
Software	BD FACSDiva Software (Version 8.0.1) was used for flow cytometry data collection. FlowJo software (v.10.5.0) was used for data analysis and presentation
Cell population abundance	Cy5+ and Cy5- sorted populations were assessed for post-sort purity by flow cytometry and anti-Cy5 immunostaining. For anti-cy5 immunostaining flow sorted sputum samples were spread across Superfrost Plus microscope slides using a sterile pipette tip and allowed to air dry for 30 min. Slides were washed 3X in PBS and blocked using 1% goat serum in PBS for 1h, followed by treatment with an anti-Cy5 monoclonal antibody (C1117, Sigma-Aldrich) (1:100 dilution) in incubation buffer (1% goat serum, 0.3% Triton X100 and 10mg/mL bovine serum albumin) overnight at 4C. Slides were washed 3X, and incubated with Cy3 goat anti-mouse secondary antibody (1:250) in incubation buffer for 45 min. Slides were washed 2X, counterstained using 0.1% Hoescht 33342 in PBS and mounted using Prolong Diamond Antifade.<br>Slides were imaged using an Olympus IX83 microscope with a transmitted Koehler illuminator and a 60X oil objective lens (NA 1.42). Images were captured on a Hamamatsu ORCA-Flash4.0 V2 digital CMOS camera, and post-acquisition image analysis was performed using cellSens software (v.1.14, Olympus).<br>Image analysis was performed using FIJI (59). Briefly, images were subjected to background subtraction using a rolling ball radius of 150 pixels. Individual cells were identified by adjusting thresholds of SYTO64 images using Huang's fuzzy thresholding method (60). Images were also segmented using a watershedding algorithm that assumes each maximum belongs to a discrete particle. The 'Analyze Particles' operation was used to detect and record locations of individual bacterial cells in a given image. For clinical samples, particles were constrained between 100-1000 pixels to minimize detection of host cells and sputum debris. Mean pixel intensity at 546nm (Cy3-labeled anti-Cy5) was then quantified for each assigned particle. Imaging experiments were performed in triplicate for each bacterial species, and ten images for each sample were captured (n>1000 particles per sample).

Gating strategy

AHA- control samples were first gated based on PE anti-human CD45RO staining to remove human leukocytes. Next, the negative control was used to measure background Cy5 fluorescence to define Cy5+ (i.e. active) and Cy5- (i.e. inactive) sorting gates. A patient matched AHA+ sample was gated using PE anti-human CD45RO, followed by Cy5+ and Cy5- gates based on the patient-matched AHA- control. Lastly, forward scatter and side scatter gates were applied to remove large particulates and debris, and liberal doublet discrimination was used to minimize loss of bacterial aggregates.

☒ Tick this box to confirm that a figure exemplifying the gating strategy is provided in the Supplementary Information.
